# CO_2_ Hydrogenation to Methanol over Mesoporous SiO_2_-Coated Cu-Based Catalysts

**DOI:** 10.1021/acsnanoscienceau.4c00016

**Published:** 2024-07-18

**Authors:** Luiz H. Vieira, Marco A. Rossi, Letícia F. Rasteiro, José M. Assaf, Elisabete M. Assaf

**Affiliations:** † São Carlos Institute of Chemistry, University of São Paulo, São Carlos, São Paulo 13560-970, Brazil; ‡ School of Chemical & Biomolecular Engineering, Georgia Institute of Technology, Atlanta, Georgia 30332, United States; § Department of Chemical Engineering, Federal University of São Carlos, São Carlos, São Paulo 13565-905, Brazil

**Keywords:** CO_2_ utilization, methanol synthesis, copper, indium, silica, core−shell, mesoporous material

## Abstract

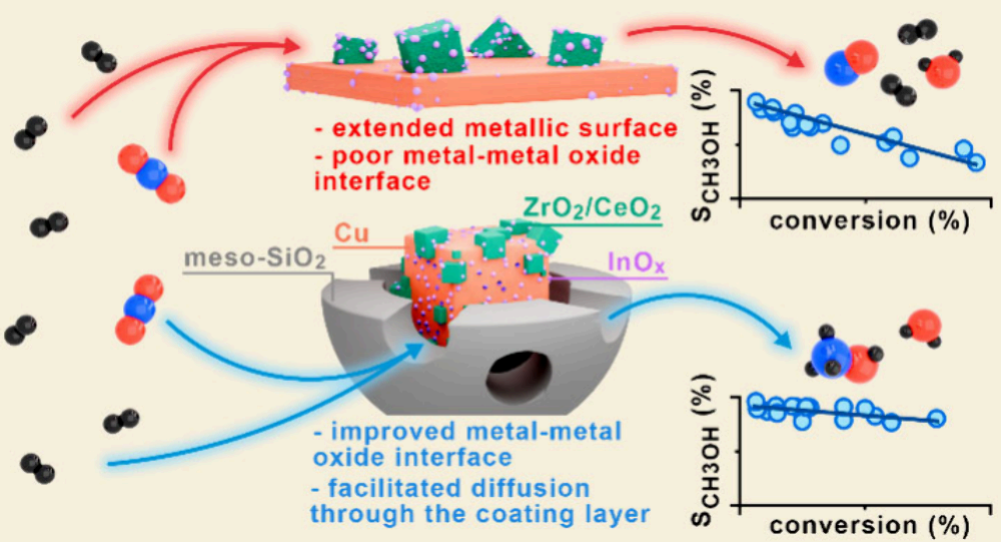

Although chemical promotion led to essential improvements in Cu-based catalysts for CO_2_ hydrogenation to methanol, surpassing structural limitations such as active phase aggregation under reaction conditions remains challenging. In this report, we improved the textural properties of Cu/In_2_O_3_/CeO_2_ and Cu/In_2_O_3_/ZrO_2_ catalysts by coating the nanoparticles with a mesoporous SiO_2_ shell. This strategy limited particle size up to 3.5 nm, increasing metal dispersion and widening the metal–metal oxide interface region. Chemometric analysis revealed that these structures could maintain high activity and selectivity in a wide range of reaction conditions, with methanol space-time yields up to 4 times higher than those of the uncoated catalysts.

Methanol is a versatile chemical compound that finds applications in various industries, including fuel production and chemical synthesis, and it is used as a feedstock for other chemicals.^1^ Traditionally, methanol has been primarily produced from natural gas or coal through the syngas route.^2^ However, as the world focuses on reducing greenhouse gas emissions and transitioning to more sustainable practices, researchers have been exploring alternative methods. Renewable methanol production from CO_2_ hydrogenation holds promise as a viable pathway to achieving these goals.^3,4^ CO_2_ can be sourced from various industrial processes, such as power plants, cement manufacturing, or even directly from the atmosphere using carbon capture technologies.^5−7^ Looking for net zero carbon emissions, the H_2_ required for this reaction can be obtained through water electrolysis, utilizing renewable energy sources such as wind or solar power.^8,9^

The conventional methanol industry relies on Cu/ZnO/Al_2_O_3_ catalysts due to their low cost and high activity. However, when directly converting CO_2_ to methanol, this catalyst faces challenges like low single-pass conversion, low methanol selectivity, high-pressure requirements, and fast deactivation.^10^ Based on this, recent research has focused on improving activity and stability by modifying existing catalysts and developing new ones. Cu/ZrO_2_^11−15^ and Cu/CeO_2_^15−19^ catalysts have appeared as alternatives to improve the reaction. The unique electronic properties generated in the metal–metal oxide interfaces of these catalysts promote the adsorption and activation of reactants and intermediates^20^ when related to unsupported Cu catalysts.^21,22^ These catalysts are highly selective to methanol at relatively low temperatures, but their kinetics limits the conversion rates. The drop in selectivity by increasing temperature is notable due to competing endothermic reverse water–gas shift (rWGS) reaction.^23^ The aggregation of Cu particles at these conditions results in weak CO adsorption sites, making the intermediate to desorb as the major product.^24^ Chemical promotion of Cu/ZrO_2_ and Cu/CeO_2_ catalysts with low loadings of In_2_O_3_ emerged as a strategy to increase the metal–support interaction, enhancing dispersion and decreasing and stabilizing Cu nanoparticles.^25−30^ Although this system has shown promise for CO_2_ hydrogenation, it still suffers from decreasing CH_3_OH selectivity by gradually increasing reaction temperature, indicating that there is room for further improvements.

Based on this, improvements in the physical properties of these catalysts were explored in this work. We coated hydrothermally synthesized Cu/In_2_O_3_/CeO_2_ and Cu/In_2_O_3_/ZrO_2_ nanoparticles with a mesoporous SiO_2_ shell, prepared using CTAB and TEOS as precursors (See complete method description in Supporting Information). The catalysts were named CuCeIn@mSiO_2_ and CuZrIn@mSiO_2_, respectively, and were compared to reference uncoated materials named CuCeIn and CuZrIn. The SiO_2_ shell accounts for 80 wt % of the catalyst composition for coated materials. The core composition was close to the expected nominal values of 50 mol % Cu, 45 mol % Ce/Zr, and 5 mol % In for both catalysts (Table 1).

**Table 1:** Physical and Chemical Properties of SiO_2_-Coated and Uncoated CuZrIn and CuCeIn Catalysts

	Composition (mol %)^a^									
Catalyst	Ce	Zr	Cu	In	Surface area (m^2^·g^–1^)	Mesopore volume (cm^3^·g^–1^)	Mesopore diameter (nm)	Cu dispersion (%)	Metallic surface area (m_Cu_^2^.g_cat_^–1^)	Basicity (μmol_CO2_·g^–1^)
CuCeIn@mSiO_2_	42.5	0	53.1	4.4	192	0.59	5.4	22.7	14.6	288
CuCeIn	40.5	0	54.7	4.8	46	0.05		6.5	10.5	242
CuZrIn@mSiO_2_	0	43.1	52.3	4.6	235	0.95	5.4	29.6	21.1	194
CuZrIn	0	57.1	39.2	3.7	29	0.03		10.0	20.3	126

a Compositions of CuCeIn@mSiO_2_ and CuZrIn@mSiO_2_ are related to CuCeIn and CuZrIn cores, respectively. These materials present 80% of SiO_2_ and 20% of other elements (Cu, Zr, Ce and In).

Regarding structural properties, XRD patterns (Figure 1a) revealed peaks of fluorite-type cubic CeO_2_^31^ and CuO^32^ structures in the CuCeIn sample and only CuO-related peaks in the CuZrIn, probably due to the amorphous ZrO_2_ precipitation. The coated materials, CuCeIn@mSiO_2_ and CuZrIn@mSiO_2_, have shown patterns characteristic of amorphous silica material where peaks related to CeO_2_ and ZrO_2_ phases are barely seen. As reported by some authors,^33−36^ the lower aggregation of confined core particles during the heat treatment keeps their dimensions smaller than those formed in uncoated materials. Since particle size is directly related to crystalinitty,^37^ XRD provides the primary qualitative evidence for forming small active phase cores resistant to aggregation. The textural properties of the catalysts were accessed through N_2_ adsorption–desorption isotherms (Figure 1b). As expected, the BET surface area of catalysts considerably increased (Table 1), mainly due to the mesoporosity generated by the presence of the SiO_2_ shell. CuCeIn@mSiO_2_ and CuZrIn@mSiO_2_ showed pore volumes of 0.59 and 0.95 cm^3^·g^–1^, respectively, which was in the range of SBA-15 (0.80–1.00 cm^3^·g^–1^),^38^ a long-range ordered mesoporous silica prepared using the same surfactant. Since the pore volume combines contributions of intra- and interparticle SiO_2_ shell porosity and, considering the identical procedure for intraparticle mesoporosity generation applied during core coating and the close core diameters in both CuZrIn@mSiO_2_ and CuCeIn@mSiO_2_ catalysts, the difference in pore volume probably arises from interparticle contribution, due to distinct SiO_2_ aggregation. The pore size distributions (Figure 1c) revealed a relatively narrow distribution of mesopores around 5.4 nm in diameter, which indicates that diffusion of reagents and products (kinetic diameters in the range 0.28–0.36 nm^39,40^) to and from active sites should not be affected during the reaction.

**Figure 1:**
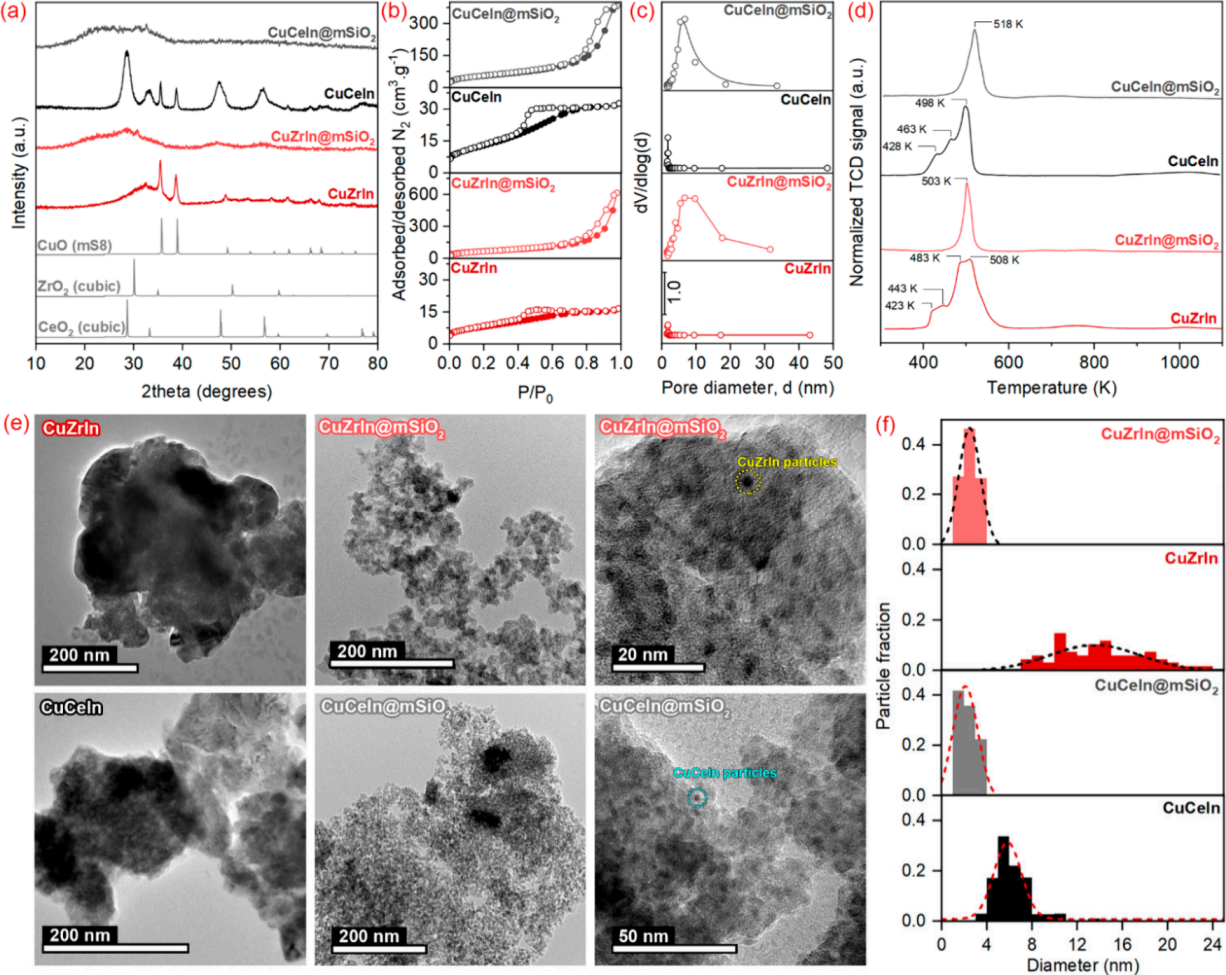
(a) X-ray diffraction patterns, (b) N_2_ isotherms, (c) pore size distribution, (d) temperature-programmed reduction profiles, (e) transmission electron microscopy images, and (f) particle size distribution of SiO_2_-coated and uncoated CuZrIn and CuCeIn catalysts.

The reducibility of catalysts was monitored by TPR under H_2_ stream_,_ and the obtained profiles are shown in Figure 1d. Intense peaks between 420 and 520 K were generally verified for all materials, a characteristic temperature range for reducing CuO to Cu^0^.^41^ SiO_2_-coated materials presented a single narrow peak, while convoluted peaks were noted for the uncoated ones. The multiple peaks are associated with CuO particles with distinct physicochemical properties such as size, dispersion, and degree of interaction with other components.^42^ In this way, the number of peaks is generally proportional to the homogeneity of the particle diameter. Thus, the peaks that appear at lower temperature values would be associated with smaller particles presenting a high surface-area-to-volume ratio, while the high-temperature peaks are related to larger particles. The single peak in CuZrIn@mSiO_2_ and CuCeIn@mSiO_2_ profiles indicates the high homogeneity in particle size, and the slight shift to higher temperatures is probably due to the difficulted heat-transfer from the thick SiO_2_ shell to copper in the core nanoparticles, since the heating rate was kept constant in all experiments. The TEM images (Figure 1e) and particle size distribution (Figure 1f) corroborate previous characterizations. CuZrIn and CuCeIn catalysts have shown aggregated and less homogeneous particles. Particularly, CuZrIn showed a broad distribution compared to CuCeIn. The specific elements involved (Cu, Zr, and In) might interact differently during the catalyst formation, leading to a broader range of particle sizes. These interactions can affect the crystallization process and the final size distribution. At the same time, the higher contrast clearly shows the presence of cores around 1.5–3.5 nm in CuZrIn@mSiO_2_ and CuCeIn@mSiO_2_ catalysts. It is important to note that particle sizes of coated catalysts are related to a core combining metal and metal oxides, while particle sizes indicated in Figure 1f for uncoated catalysts are related to ZrO_2_ and CeO_2_ particles. Our previous works indicated that CuO domains in uncoated catalysts are around 25 and 15 nm, growing to 65 and 30 nm after reduction to metallic Cu, for CuCeIn and CuZrIn catalysts, respectively.^29,30^

The metallic surface area of catalysts (Table 1) was accessed through the H_2_ consumption related to the reduction of the surface CuO layer, previously generated by controlled oxidation under an N_2_O stream.^43^ It is possible to note that the dispersion of copper atoms in the CuZrIn@mSiO_2_ and CuCeIn@mSiO_2_ catalysts was around 3–4 times higher than that observed for the pristine samples, which can be explained in terms of the efficiency of the solvothermal method in generating well-dispersed particles, as well as the functionality of the SiO_2_ coating in preventing particle aggregation during thermal treatments.^44,45^ Despite the notably higher dispersion, the increase in the metallic surface area (Table 1) was not proportional to differences in catalyst composition. SiO_2_ is the most abundant compound within coated materials, and the active phase content (Cu, InO_*x*_, ZrO_2,_ and CeO_2_) constitutes only a tiny fraction of the catalyst compared to uncoated ones. Normalizing the metallic surface area by the unit mass of active compounds (Table S1) makes the improvement in the coated materials evident. These catalysts also demonstrated higher basicity, evaluated through the CO_2_ chemisorption capacity (Table 1). It can be attributed to the smaller average particle size, which can ensure a higher density of oxygen vacancies that act as strong Lewis basic sites.^46^ Additionally, CuCeIn@mSiO_2_ exhibited higher basicity than CuZrIn@mSiO_2_, which is expected given the inherent high basicity of lanthanides due to their propensity for electron donation.^47^ Considering that the chemical nature of the coating layer prevents significant interaction of CO_2_ molecules, only the active phase of the coated catalysts is responsible for the basicity, thus being significantly superior to that of uncoated catalysts (Table S1).

To effectively demonstrate the improvement in catalytic performance, the intrinsic activity of SiO_2_-coated and uncoated materials was compared using the turnover frequency (TOF), as shown in Table 2. Although the metal–metal oxide interface is crucial for binding CO_2_ and facilitating reaction,^20,22^ hydrogenation steps are generally the determining steps.^48,49^ Therefore, we employed a metallic surface area as the active site for TOF calculations. Additionally, the affinity of CeO_2_ and ZrO_2_ surfaces with CO_2_ results in the formation of passive surface carbonate species, making it challenging to distinguish them from active species through CO_2_-TPD analysis. It is well-known from the literature that methanol production from CO_2_ hydrogenation can follow mainly two competitive routes: (1) formate and (2) rWGS + CO hydrogenation.^50^ In the latter case, depending on the interaction between CO and the catalyst surface, CO can either be desorbed as a product or further hydrogenated to form methanol. Comparing CuCeIn@mSiO_2_ and CuCeIn catalysts, a substantial increase in the total TOF is noted, from 2.12 × 10^–3^ to 3.43 × 10^–3^ s^–1^, which means an increase in the catalyst efficiency for the coated catalysts. A substantial increase was also noted for the TOF of methanol and a decrease in the TOF of CO. Since both routes (rWGS and formate) compete during the process, this result indicates that the CO production by the rWGS reaction is effectively suppressed in the coated catalyst, favoring methanol production. The total TOF of CuZrIn@mSiO_2_ and CuZrIn remains similar (∼3.1 × 10^–3^ s^–1^), but the specific rate calculated for CO production from rWGS reaction decreased from 0.93 to 0.14 × 10^–3^ s^–1^ while the TOF for methanol production proportionally increased. The strong metal–support interaction (SMSI) arising from Cu and amorphous ZrO_2_ interface^14^ partially inhibits nanoparticle aggregation, even in uncoated catalysts, explaining the closely matched total TOF values. Furthermore, in coated material, the physical constraints add stability and promote the maintenance of smaller particles and, consequently, higher metal dispersion, justifying the changes in specific TOF values for CO and methanol formation.

**Table 2:** Turnover Frequency (TOF) and Apparent Activation Energies for rWGS and Methanol Synthesis over Uncoated and SiO_2_-Coated CuCeIn and CuZrIn Catalysts

	TOF (10^–3^ s^–1^)^a^			Apparent activation energy (kJ mol^–1^)	
Catalyst	CO (rWGS)	CH_3_OH	Total (CO + CH_3_OH)	CO (rWGS)	CH_3_OH
CuCeIn@mSiO_2_	0.13	3.30	3.43	105.0	37.2
CuCeIn	0.81	1.31	2.12	116.3	43.1
CuZrIn@mSiO_2_	0.14	2.94	3.08	92.9	32.6
CuZrIn	0.93	2.23	3.16	97.1	36.0

a Reaction conditions for TOF calculation: *T* = 498 K, *P* = 2.5 MPa and WHSV = 14000 mL·g^–1^·h^–1^, *X*_CO2_ ≤ 4.2%.

The apparent activation energies (Table 2) for CO production by rWGS and methanol production were calculated for the catalysts using Arrhenius plots (Figure S1). Analyzing these values (Table 2), we observe a decrease in the activation energy for both rWGS and methanol formation in coated catalysts. However, the rWGS energy barrier remains significantly higher than that for hydrogenation route for all evaluated catalysts. This observation suggests that the chemical nature of the active site is likely consistent between coated and uncoated catalysts. It confirms that the mesoporous SiO_2_ only acts to prevent surface restructuring due to aggregation. As a result, interfacial active sites for hydrogenation are preserved, and extensive metallic surfaces in larger Cu particles that promote the reverse water–gas shift (rWGS) reaction^24^ are avoided. In general, it can be said that the physical barriers created only regulate the ratio between the active sites for CH_3_OH formation and those active for CO formation, favoring the maintenance of the former when the catalyst is applied to the reaction environment.

To gain information related to catalyst behavior on relevant reaction conditions for methanol synthesis from CO_2_, we conducted a chemometric analysis^51^ using a central composite experimental design (fully described in the Supporting Information) for both coated and uncoated materials. Based on reaction conditions commonly reported in the literature for Cu-based materials, the temperature, pressure, and WHSV ranges were defined as described in Table S2, resulting in an experimental matrix composed of 17 reaction conditions (Table S3). The results achieved by the proposed experimental matrix regarding CO_2_ conversion, CH_3_OH selectivity, and space-time yield (STY) are shown in Figure 2 and Tables S4–S7. A general improvement was observed in most of the reaction conditions tested, but it becomes clear that using SiO_2_-coated materials is advantageous precisely under high temperatures (≥523 K) in which the aggregation of particles becomes more intense in former catalysts. Although some increase in the CO_2_ conversion was achieved in coated catalysts, the high CH_3_OH selectivity is what truly distinguishes these catalysts, which led to productivities of 268 mg_CH3OH_.g_cat_^–1^·h^–1^ (89% selectivity) for CuCeIn@mSiO_2_ and 345 mg_CH3OH_.g_cat_^–1^·h^–1^ (83% selectivity) for CuZrIn@mSiO_2_ at 523 K and 3.0 MPa, while the uncoated catalysts achieved maximum productivities of 77 mg_CH3OH_.g_cat_^–1^·h^–1^ (42% selectivity) for CuCeIn and 233 mg_CH3OH_.g_cat_^–1^·h^–1^ (52% selectivity) for CuZrIn at the same conditions. These results point to a satisfactory development when compared to most Cu-based catalysts used in the production of CH_3_OH presented in the literature (Table S8). The synergy between three components (Cu, In_2_O_3,_ and CeO_2_/ZrO_2_) is crucial to keep high activity since SiO_2_-coated catalysts containing one (Cu@SiO_2_, In_2_O_3_@SiO_2_) or two components (Cu/ZnO@SiO_2_, Cu/In_2_O_3_@SiO_2_)^35,36^ in core presented productivities in the range 0.07–0.21 g_CH3OH_.g_cat_^–1^·h^–1^. Additionally, the very low loadings of In in coated catalysts (∼1 wt %) can make these materials more attractive economically.

**Figure 2:**
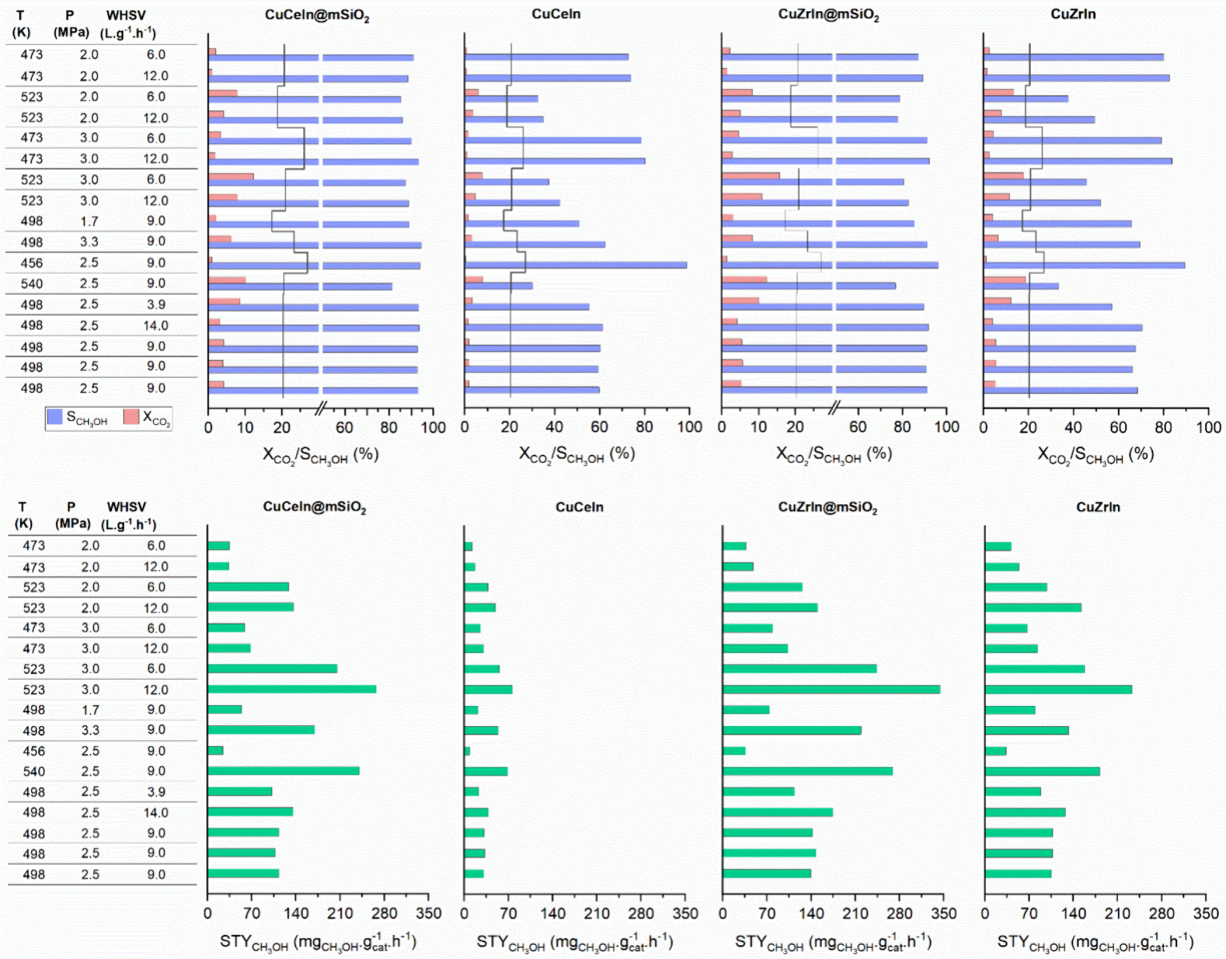
Catalytic results from the experimental design matrix defined by the central composite methodology. The black lines in the graphs indicate the equilibrium conversion for the respective reaction conditions applied. Catalysts were tested three times at central point reaction conditions (498 K, 2.5 MPa and 9 L·g^–1^·h^–1^) to check the reproducibility of the experimental setup.

The effects caused by isolated variables (T, P, and WHSV) or by the combination of these variables were calculated, and their significance was statistically evaluated based on CH_3_OH selectivity (Tables S9–S12), CO_2_ conversion (Tables S13–S16) and CH_3_OH productivity (Tables S17–S20). The standardized effects are illustrated in Pareto’s charts in Figures S2–S4. First, it is essential to note that the effect of isolated variables is relatively more significant than the combined effect, ensuring that a separate analysis of each variable is relevant. CuCeIn exhibits the highest sensitivity to the reaction temperature. In contrast, this effect is less pronounced in CuZrIn, likely due to the particle resistance resulting from the strong metal–support interaction (SMSI), as mentioned earlier. The SiO_2_ coating has significantly reduced the materials’ sensitivity to temperature, while increasing pressure seems to have a more significant positive impact on selectivity. This phenomenon can be attributed to the confined environment in which the active sites are situated. As the increase in CO_2_ conversion is favored by increasing temperature, as expected, the maximum methanol productivity will be achieved based on these materials’ ability to maintain selectivity at higher temperatures.

Aiming to project selectivity, conversion, and productivity values for specific combinations of pressure, temperature, and space velocity that were not tested within the experimental matrix defined by the central composite design, we constructed response surfaces by using a quadratic model shown in Figure 3. This model involves fitting the experimental data to a second-degree polynomial equation (standard form y = ax^2^ + bx + c). The equations that originated the surfaces are described in the Supporting Information (eqs S6–S17). The analysis of variance (ANOVA, Tables S21–S32) and the comparison between predicted and experimental results (Figure S5) indicates that around 90% of the total variation in the responses is adequately explained using regression equations generated by the quadratic model, which is confirmed by R^2^ values. A crucial observation is that in general, the effects of each variable on CH_3_OH selectivity for the coated catalysts were considerably smaller than those in the former catalysts. As also mentioned for experimental data, this difference is particularly noticeable regarding the temperature effect, which implies that SiO_2_-coated catalysts exhibit lower sensitivity to variations in reaction conditions, especially temperature, maintaining high methanol selectivity, even under the conditions where rWGS is the main route for uncoated materials. CuCeIn@mSiO_2_ exhibited slightly less sensitivity to selectivity loss with temperature compared to CuZrIn@mSiO_2_. However, due to its slightly lower CO_2_ conversions, the maximum predicted productivity is around 400 mg_CH3OH_.g_cat_^–1^·h^–1^, while for CuZrIn@mSiO_2_ is approximately 500 mg_CH3OH_.g_cat_^–1^·h^–1^. Despite these differences, it is crucial to note that adjustments in reaction conditions should allow for relatively higher productivities than those achieved under the tested experimental conditions while maintaining high selectivities for CH_3_OH (70–80%). The statistical confirmation of the low impact of temperature on hydrogenation in coated catalysts aligns well with previously reported works regarding the potential of mesoporous SiO_2_ to minimize particle aggregation under thermal treatments and reaction conditions.^52^ This approach yields promising results, especially when conventional catalysts experience a considerable decline in their performance.

**Figure 3:**
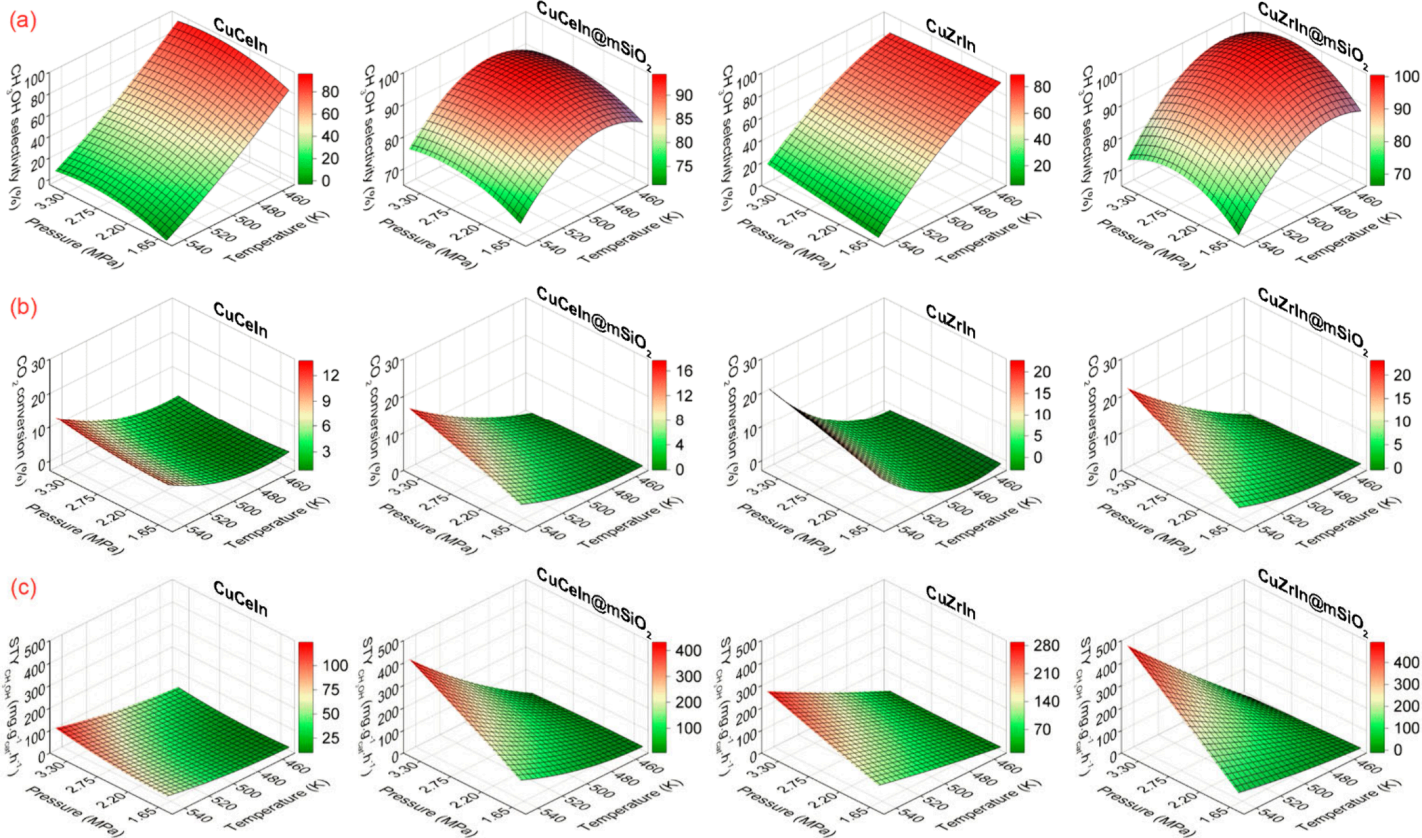
Surfaces built for uncoated and coated catalysts showing the responses of (a) CH_3_OH selectivity, (b) CO_2_ conversion, and (c) CH_3_OH space-time yield (STY) during hydrogenation by varying the reaction temperature and pressure and keeping WHSV = 12 L·g^–1^·h^–1^.

To evaluate the stability and confirm the high productivities at harsh conditions, CuZrIn@mSiO_2_ and CuCeIn@mSiO_2_ catalysts were submitted to a series of reuse tests at 543 K, 3.3 MPa and variable WHSV values (6, 9, and 12 L·g^–1^·h^–1^) as shown in Figure S6. The catalysts achieved the expected CH_3_OH productivities in the range 400–500 mg_CH3OH_.g_cat_^–1^·h^–1^ with selectivities higher than 70% in space velocities of 12 L·g^–1^·h^–1^ as predicted by surface responses in Figure 3. After six reuses, the high catalytic activity persisted. Additionally, the spent materials were characterized by XRD, TEM and EDS analysis. No crystalline Cu phase was identified in diffraction patterns (Figure S7), qualitatively evidencing the maintenance of the nanoparticle size. The TEM images, particle size distribution (Figure S8), and elemental mappings (Figure S9) quantitatively confirmed that cores kept their sizes smaller than 4 nm and active phase kept homogeneously dispersed in catalysts.

In summary, our investigation focused on the impact of confining Cu/In_2_O_3_/ZrO_2_ and Cu/In_2_O_3_/CeO_2_ nanoparticles, by adding a mesoporous SiO_2_ coating layer, in the CO_2_ hydrogenation. This approach successfully limited nanoparticle growth, resulting in cores with sizes of up to 3.5 nm. By constructing response surfaces through varying reaction conditions using a statistical approach based on an experimental matrix, we identified that the temperature’s effect on reducing selectivity is significantly diminished in SiO_2_-coated catalysts, leading to high CH_3_OH productivity. Furthermore, preventing metallic surface agglomeration suppressed competition with the rWGS reaction at higher temperatures. Our work proposes a strategy to enhance the physical properties of Cu-based catalysts, providing new insights into catalyst design for methanol production from CO_2_.

## Supporting Information Available

Description of experimental methods and supplemental data related to the characterization, catalytic activity and chemometric approach employed (PDF)
