# Supplementary material for: CO2 Hydrogenation to Methanol over Mesoporous SiO2-Coated Cu-Based Catalysts
Source: ACS Nanosci Au. 2024 Jul 18;4(4):235–42. doi: 10.1021/acsnanoscienceau.4c00016 (PMC11342343; doi:10.1021/acsnanoscienceau.4c00016)
Supplement: Supplementary file 1 — ng4c00016_si_001.pdf [file ng4c00016_si_001.pdf]

Supplementary Material

CO_2 hydrogenation to methanol over mesoporous SiO_2-coated Cu-based catalysts

Luiz H. Vieira[1]*, Marco A. Rossi[1], Letícia F. Rasteiro[2], José M. Assaf[3], Elisabete M. Assaf[1]*

[1]*São Carlos Institute of Chemistry, University of São Paulo, São Carlos, São Paulo, 13560-970, Brazil*

[2]*School of Chemical & Biomolecular Engineering, Georgia Institute of Technology, Atlanta, Georgia 30332, United States*

[3]*Department of Chemical Engineering, Federal University of São Carlos, São Carlos, São Paulo, 13565-905, Brazil*

*Corresponding authors: eassaf@iqsc.usp.br; lhvieira@iqsc.usp.br

KEYWORDS: CO_2 utilization; methanol synthesis; copper; indium; silica; core-shell; mesoporous material.

1. Experimental Section

1.1. Synthesis of In-promoted catalysts

The catalysts were synthesized using the surfactant-assisted co-precipitation method. Initially, 60 mmol of CTAB were added to 250 mL of H_2O. The solution was kept under constant stirring until complete homogenization. Then, 15 mmol of $Cu(NO_3)_2$, 13.5 mmol of $ZrO(NO_3)_2$ or $Ce(NO_3)_3$, and 1.5 mmol of $In(NO_3)_3$ were added and the stirring was maintained for another 2 hours. The precipitation occurred by the addition of a 0.5 M solution of NaOH, keeping the pH at a constant value of 10 throughout the procedure. After completing the precipitation, the solution was kept under stirring for 12 hours at room temperature. The precipitate was separated by vacuum filtration and washed in H_2O until a pH ~7 was obtained in the washing solution. The solid was dried in an oven at 333 K for 48 hours and calcined at 873 K for 2 hours under an oxidizing atmosphere. The catalyst prepared using the $ZrO(NO_3)_2$ precursor was named CuZrIn and those prepared using $Ce(NO_3)_3$ were named CuCeIn.

1.2. Synthesis of SiO_2-coated catalysts

For the synthesis of the SiO_2-coated catalysts, 0.89 g of the polymer polyvinylpyrrolidone (PVP10, average molar mass equal to 10000 $g.mol^{-1}$) were dissolved in 100 mL of an H_2O and CH_3CH_2OH solution 50% (v/v) along with 0.36 g of $Cu(NO_3)_2$, 0.05 g of $In_2(NO_3)_3$, and 0.31 g of $ZrO(NO_3)_2$ (or 0.59 g of $Ce(NO_3)_3$). The mixture was stirred for 4 h at room temperature to ensure the complete solubilization of the salts and the chains of the PVP polymer. After that, the resulting solution was transferred to a stainless-steel autoclave (filling ~80% of its volumetric capacity) and kept in an oven for 12 hours at 453 K. The mixture containing the oxide particles was transferred to a 1L beaker. After this, 312.8 mL of ethanol, 93.9 mL of water, 14.5 mL of 27% (v/v) NH_4OH solution, and 0.168g of the surfactant CTAB were introduced into the same beaker. This suspension was stirred for 2 hours at room temperature followed by the addition of 2.175 mL of TEOS at a rate of ~1 drop per minute. The resulting suspension was left under vigorous stirring for 24 hours at room temperature. After this step, the suspension was centrifuged, and the obtained gel was dried 353 K for 24 hours. Finally, the resulting

solid was ground and calcined at 923 K for 6 hours. The materials were named CuZrIn@mSiO_2 and CuCeIn@mSiO_2. The molar proportions between the cations were the same as the best catalysts obtained in previous stages. 50% Cu, 5% In, and 45% Zr/Ce. PVP was employed as a control agent for the size and homogeneity of the particles that would be formed and as a directing agent, along with CTAB, for the formation of the silica coating around the oxide particles formed during the solvothermal treatment.

1.3. Catalyst characterization

1.3.1. X-ray diffraction

X-ray diffraction (XRD) measurements were conducted using a Brucker Da Vinci D8 Advance diffractometer equipped with CuKα radiation (wavelength of 0.15418 nm) and a curved graphite monochromator. Data were collected over the 2θ range from 10° to 80° with a scan step of 0.02° and a counting time of 1 s. The crystalline phases were identified using Crystallographica Search Match software.

1.3.2. X-ray fluorescence

The chemical composition of the synthesized materials was analyzed using a PANalytical X-ray fluorescence spectrometer, model MiniPal4.

1.3.3. Transmission electron microscopy

The transmission electron microscopy analyses were performed using a JEOL transmission electron microscope (model 2100). The instrument was operated with a hexaboron lanthanum (LaB_6) source and an acceleration voltage of 200 kV. The samples were previously dispersed in isopropanol using an ultrasound bath and deposited on Ni grids for analysis.

1.3.4. Temperature-programmed reduction in H_2

The temperature-programmed reduction (TPR) analysis was conducted using a Micromeritics Pulse ChemSorb 2750 instrument equipped with a thermal conductivity detector (TCD). A 50 mg sample of the catalyst was loaded into the reactor and subjected to reduction while heating in a 10% H_2/Ar gas mixture at a

flow rate of 30 mL/min. The temperature ramp ranged from room temperature to 1173 K at a rate of 10 K/min.

1.3.5. N_2 physisorption

The specific surface area and pore diameter distribution of the samples were characterized using N_2 adsorption/desorption measurements conducted at liquid nitrogen temperature (77 K). The relative pressure intervals ranged from 0.001 to 0.998. The experimental setup employed a Quantachrome Nova 100 system. Prior to analysis, the samples underwent vacuum pretreatment at approximately 10×10^{-6} Pa for 2 hours at 493 K. The BET method was utilized to determine the specific surface area (S_{BET}), while the BJH method was employed to assess mesopore volume and pore diameter distribution.

1.3.6. N_2O chemisorption

N_2O chemisorption experiments were conducted using an analytical Multipurpose system equipped with a thermal conductivity detector (TCD). Approximately 200 mg of the catalysts were loaded into a U-tube reactor. The catalyst surfaces underwent cleaning at 523 K for 1 hour under an inert atmosphere, with a flow rate of 30 mL/min. After cooling to room temperature, the samples were heated to 503 K in a 10% H_2/Ar mixture with a flow rate of 30 mL/min, ramping up at 5 K/min to completely reduce the copper. Subsequently, the samples were cooled to 298 K and exposed to a 10% N_2O/He mixture for 30 minutes to oxidize the surface copper layer. Finally, a second reduction was performed at 503 K in a 10% H_2/Ar mixture with a flow rate of 30 mL/min at 5 K/min to reduce the surface copper atoms. Between each gas mixture exchange, the system was purged using N_2 at a flow rate of 30 mL/min for 30 minutes to remove the physically adsorbed molecules remaining on the surface of the catalysts. Dispersion was calculated by the ratio between the amount of copper in the surface and total amount in the catalyst. Metallic surface area was calculated by relating the number of copper atoms in the surface and the atom density in a monolayer (Na = 1.469 x 10^{19} atoms.m^{-2}).

1.3.7. Temperature-programmed desorption of CO_2

The CO_2 temperature-programmed desorption (TPD) experiments were performed using a Micromeritics Pulse ChemSorb 2750 instrument equipped with a thermal conductivity detector (TCD). 200 mg of the catalysts were loaded into a U-tube reactor for analysis. The catalyst surfaces underwent cleaning at 523 K for 1 hour under an inert atmosphere, with a flow rate of 30 mL/min. After cooling to room temperature, the samples were heated to 503 K in a 10% H_2/Ar mixture with a flow rate of 30 mL/min, ramping up at 5 K/min. Subsequently, the system was purged with N_2 at a flow rate of 30 mL/min for 30 minutes and cooled to 298 K. The catalysts were then exposed to CO_2 at a flow rate of 30 mL/min. To remove physically adsorbed CO_2 molecules, the system was purged with N_2 once more. Finally, the catalysts were heated from room temperature to 473 K at a rate of 10 K/min and maintained at this temperature until the signal in the TCD detector returned to the baseline.

1.4. Catalytic tests

The CO_2 hydrogenation reactions were carried out in a fixed-bed stainless-steel tubular reactor with dimensions of 304.3 mm in length and an internal diameter of 9.1 mm. Prior to each test, the catalyst underwent in situ pre-reduction at 503 K for 1 hour using a pure H_2 flow (at a rate of 30 mL/min) under atmospheric pressure. The reduction temperature was chosen based on the maximum high-temperature peak observed in TPR analysis to ensure that all available surface Cu sites were effectively reduced and to prevent unnecessary use of higher temperatures. Subsequently, the reactor was cooled down to the designated reaction temperature, and the gas flow was switched to an H_2/CO_2 mixture with a molar ratio of 3:1. The system was then pressurized to specified levels. Gaseous products were analyzed using a 7890A gas chromatograph (Agilent Technologies) equipped with thermal conductivity (TCD) and flame ionization (FID) detectors. The CO_2 conversion and product selectivity were calculated using the following equations:

$$Selectivity\ to\ i\ (\%) = \left(\frac{f_i A_{i,out}}{\sum f_i A_{i,out}}\right) \times 100 \qquad \text{(S1)}$$

$$CO_2 conversion\ (\%) = \left(\frac{CO_{2,in} - CO_{2,out}}{CO_{2,in}}\right) \times 100 \qquad \text{(S2)}$$

where, $A_{i,out}$ and f_i represent the chromatogram peak area and the molar calibration factor, respectively, for each component i identified at the output. $CO_{2,in}$ and $CO_{2,out}$ represent the total number of mols of CO_2 that entered the reaction and the total number of mols of CO_2 that exited, respectively.

The space-time yield of methanol ($g_{MeOH}.kg_{cat}^{-1}.h^{-1}$) was calculated based on conversion and selectivity to methanol, using the following equation:

$$\text{STY } (Space\ Time\ Yield) = \frac{X_{CO2} \times S_{CH3OH} \times F_{CO2,\,in} \times MW_{CH3OH}}{W_{cat} \times V_m} \quad \text{(S3)}$$

where X_{CO2} represents the conversion of CO_2, S_{CH3OH} is the selectivity to CH_3OH, $F_{CO2,\,in}$ is the volumetric flow rate of CO_2 ($mL.h^{-1}$), MW_{CH3OH} is the molecular weight of methanol (32 $g.mol^{-1}$), W_{cat} is the catalyst weight used (kg), and V_m is the ideal gas molar volume at standard pressure and temperature ($mL.mol^{-1}$).

The turnover frequency (TOF) of methanol was calculated by the following equation:

$$TOF_{CH3OH}(s^{-1}) = \frac{Number\ of\ molecules\ of\ \ methanol\ produced}{Time\ (s) \times Number\ of\ metallic\ Cu\ atoms} \quad \text{(S4)}$$

$$TOF_{CH3OH}(s^{-1}) = \frac{A \times \mathcal{N}a}{3600 \times S_{Cu} \times Na} \quad \text{(S5)}$$

where A represents methanol activity in $mol.g^{-1}.h^{-1}$, $\mathcal{N}a$ is Avogadro´s number (6.023×10^{23}), S_{Cu} denotes Cu metallic area in $m^2.g^{-1}$ and Na designates the number of Cu atoms in a monolayer (Na = 1.469×10^{19} $atoms.m^{-2}$). The TOF of CO was similarly calculated, based on number of CO molecules produced.

1.5. Chemometric analysis

The impact of pressure, temperature, and space velocity parameters on the CO_2 hydrogenation process was investigated using chemometric tools. The experiments followed the Central Composite Design (CCD) methodology, which included upper, lower, center, and axial points. These points were defined for each of the variables (pressure, temperature, and space velocity) and are summarized in Table S3. We examined the main effects of each variable on methanol selectivity, as well as their interactions. The results were visually represented through Pareto

charts (Figures S2-S4). To predict methanol selectivity values for specific combinations of pressure, temperature, and space velocity that were not experimentally tested within the analysis range defined by the central composite design, we constructed response surfaces using a quadratic model. This model involved fitting the experimental data to a second-degree polynomial equation. The statistical data processing was carried out using Chemoface software version 1.6.1.

Supplementary Figures

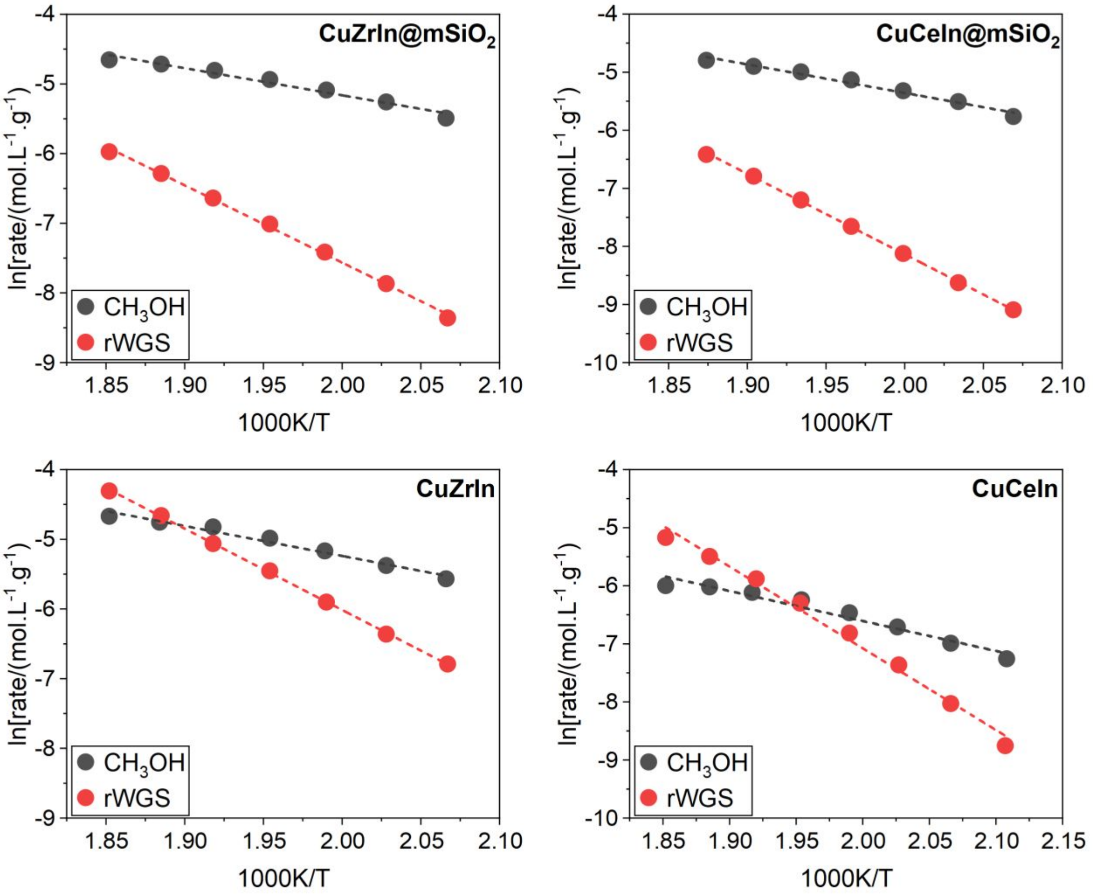

Figure S1. Arrhenius plots for CuCeIn@mSiO$_2$, CuCeIn, CuZrIn@mSiO$_2$ and CuZrIn catalysts.

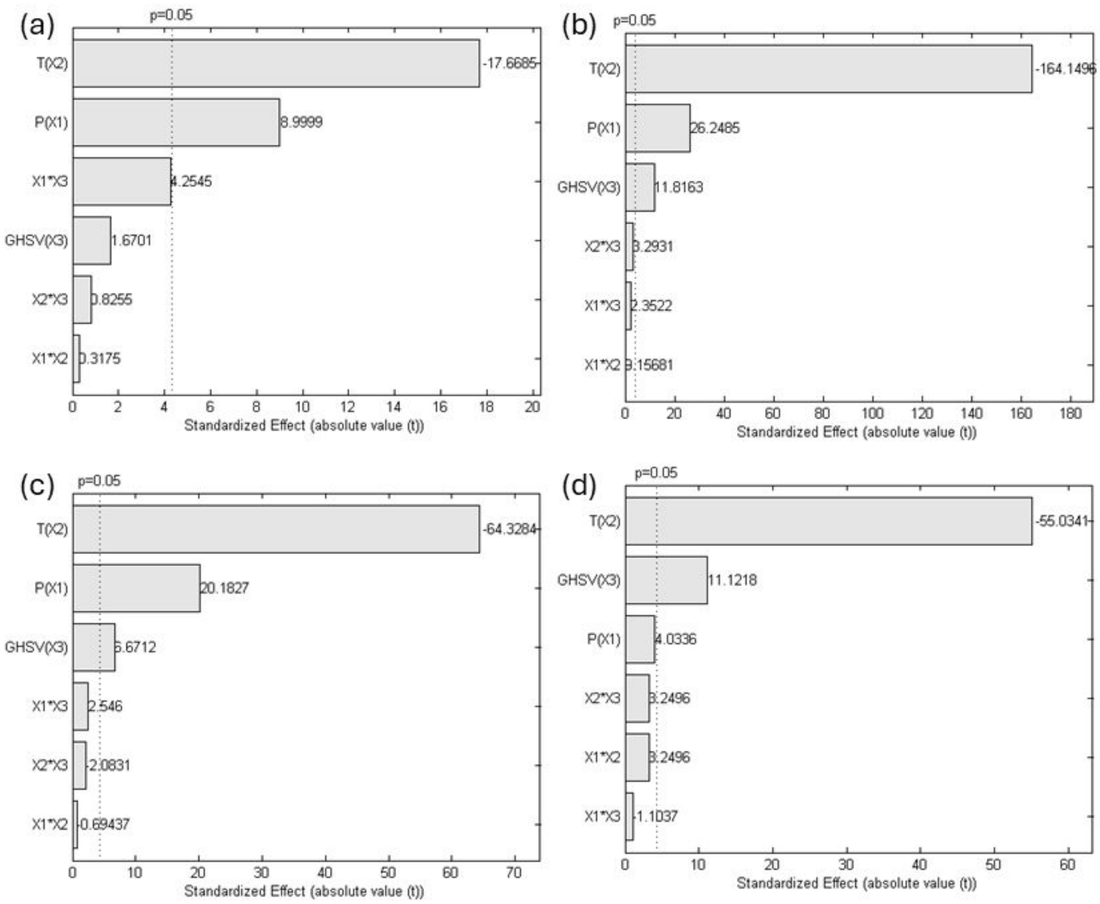

Figure S2. Pareto charts of the standardized effects caused by the variation of pressure, temperature, and space velocity on the CH_3OH selectivity for (a) CuCeIn@mSiO$_2$, (b) CuCeIn, (c) CuZrIn@mSiO$_2$ and (d) CuZrIn catalysts. The analysis was carried out with 95% confidence level.

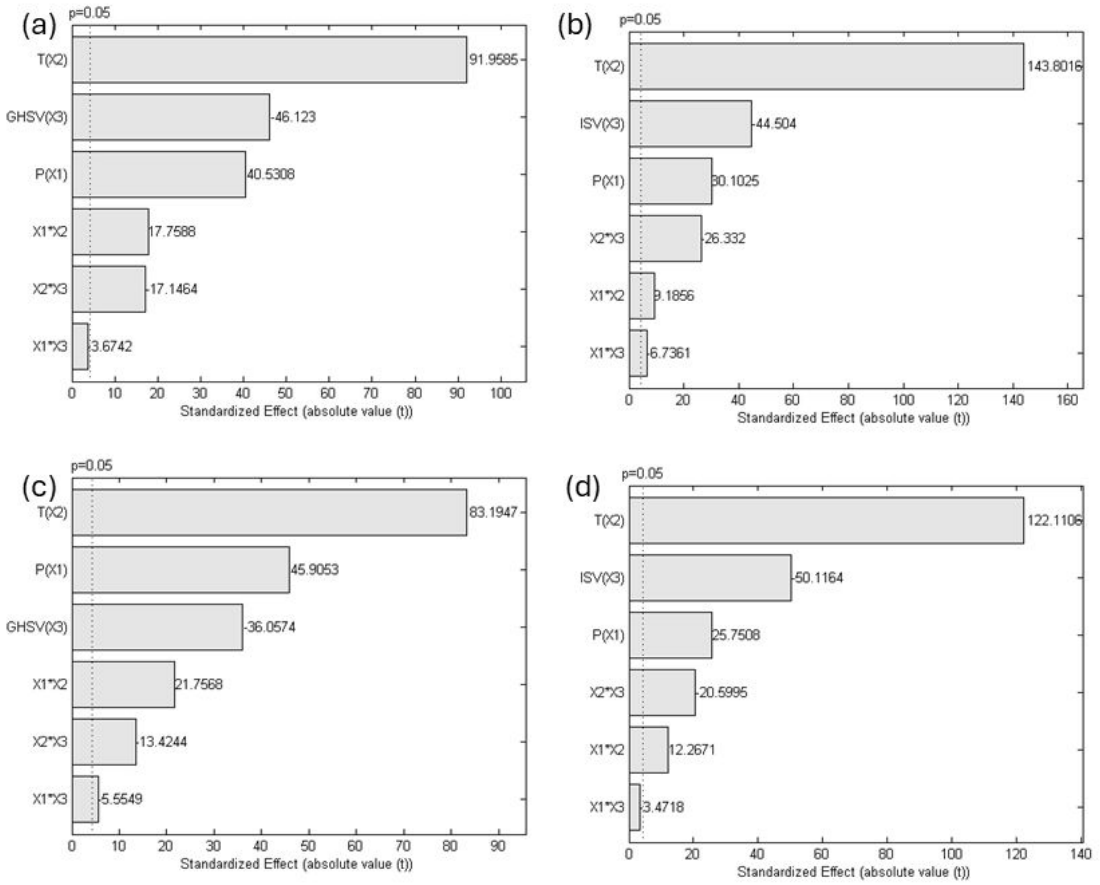

Figure S3. Pareto charts of the standardized effects caused by the variation of pressure, temperature, and space velocity on the CO_2 conversion for (a) CuCeIn@mSiO$_2$, (b) CuCeIn, (c) CuZrIn@mSiO$_2$ and (d) CuZrIn catalysts. The analysis was carried out with 95% confidence level.

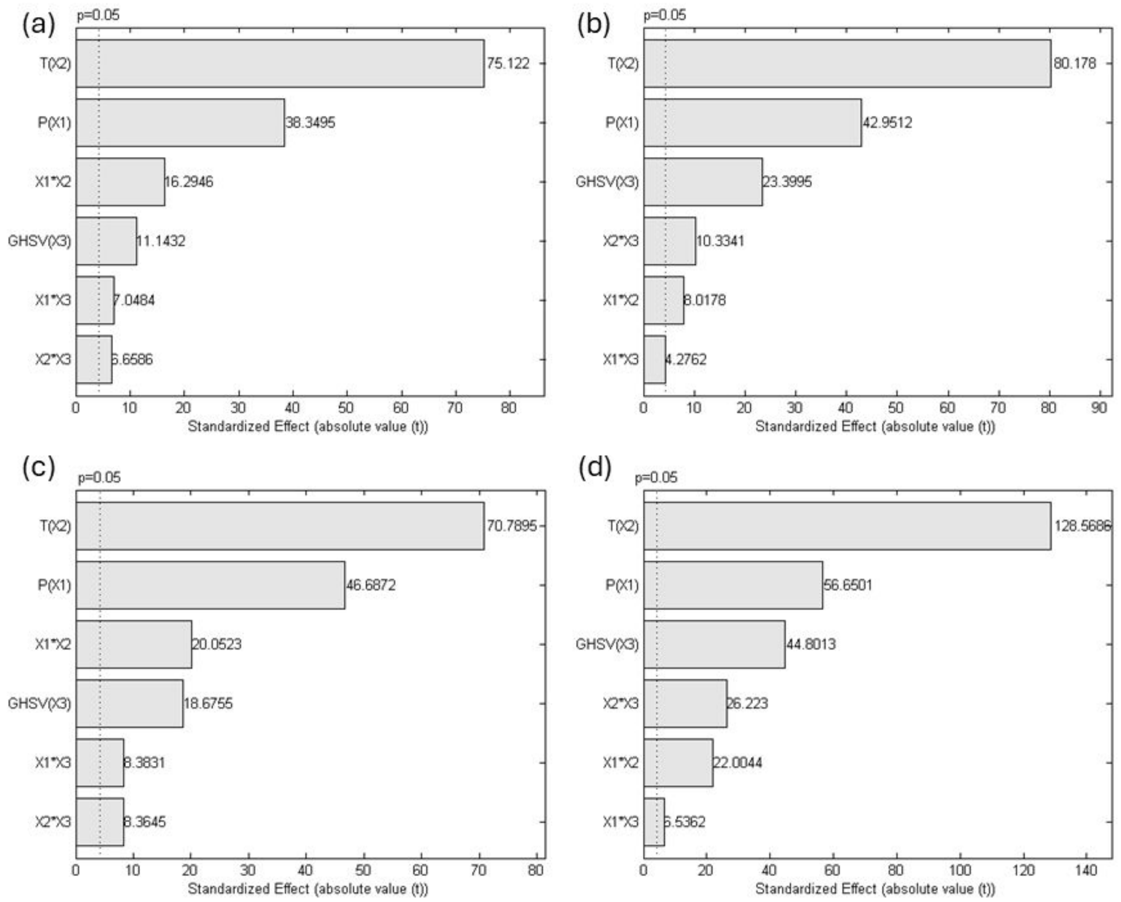

Figure S4. Pareto charts of the standardized effects caused by the variation of pressure, temperature, and space velocity on the CH_3OH space-time yield (STY) for (a) CuCeIn@mSiO_2, (b) CuCeIn, (c) CuZrIn@mSiO_2 and (d) CuZrIn catalysts. The analysis was carried out with 95% confidence level.

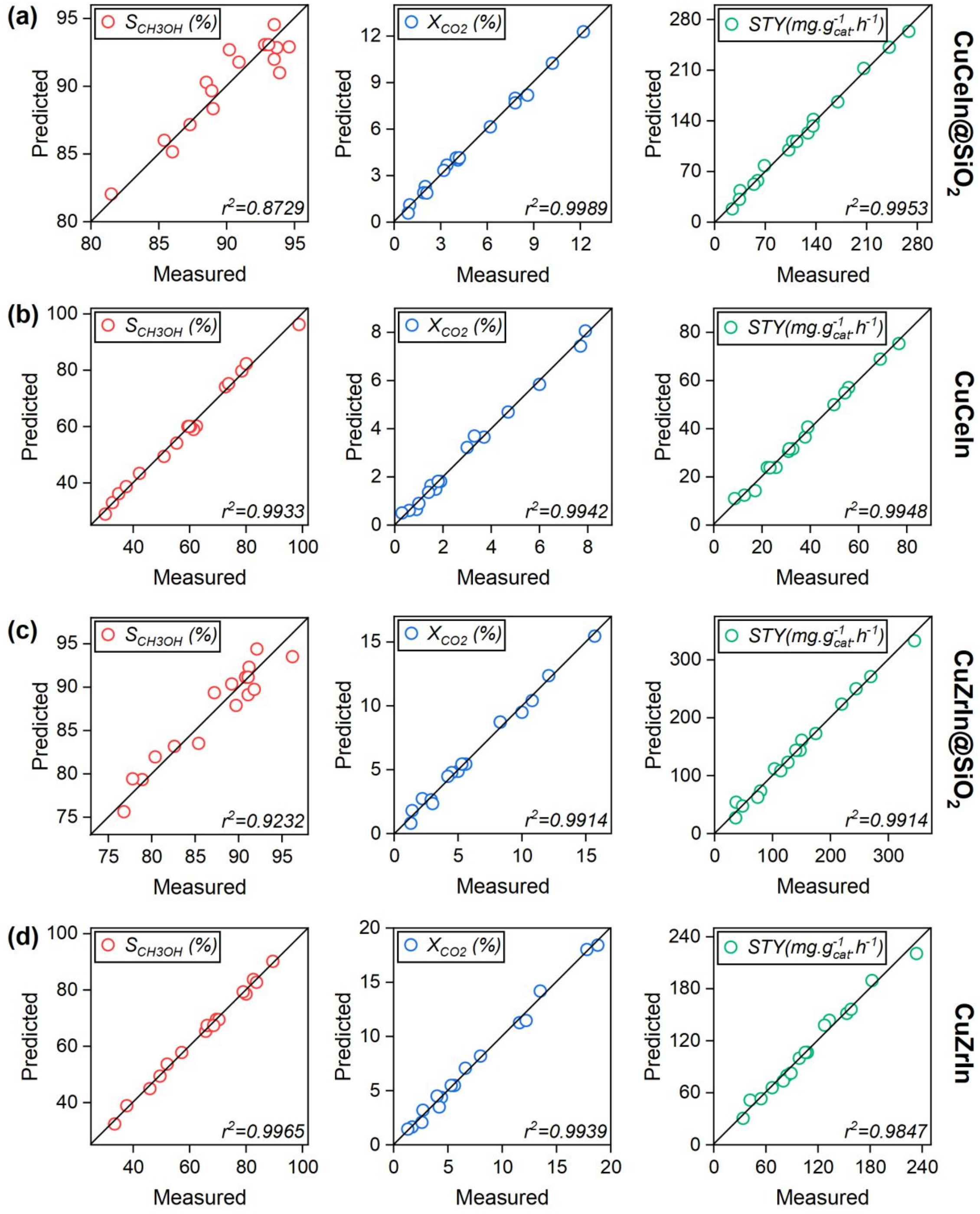

Figure S5. Comparison between predicted and measured values of CH_3OH selectivity, CO_2 conversion and CH_3OH space-time yield for (a) CuCeIn@mSiO_2, (b) CuCeIn, (c) CuZrIn@mSiO_2 and (d) CuZrIn catalysts. The predicted values were obtained by adjusting a regression equation based on the quadratic model. The experimental conditions applied are the same as described in Figure 2.

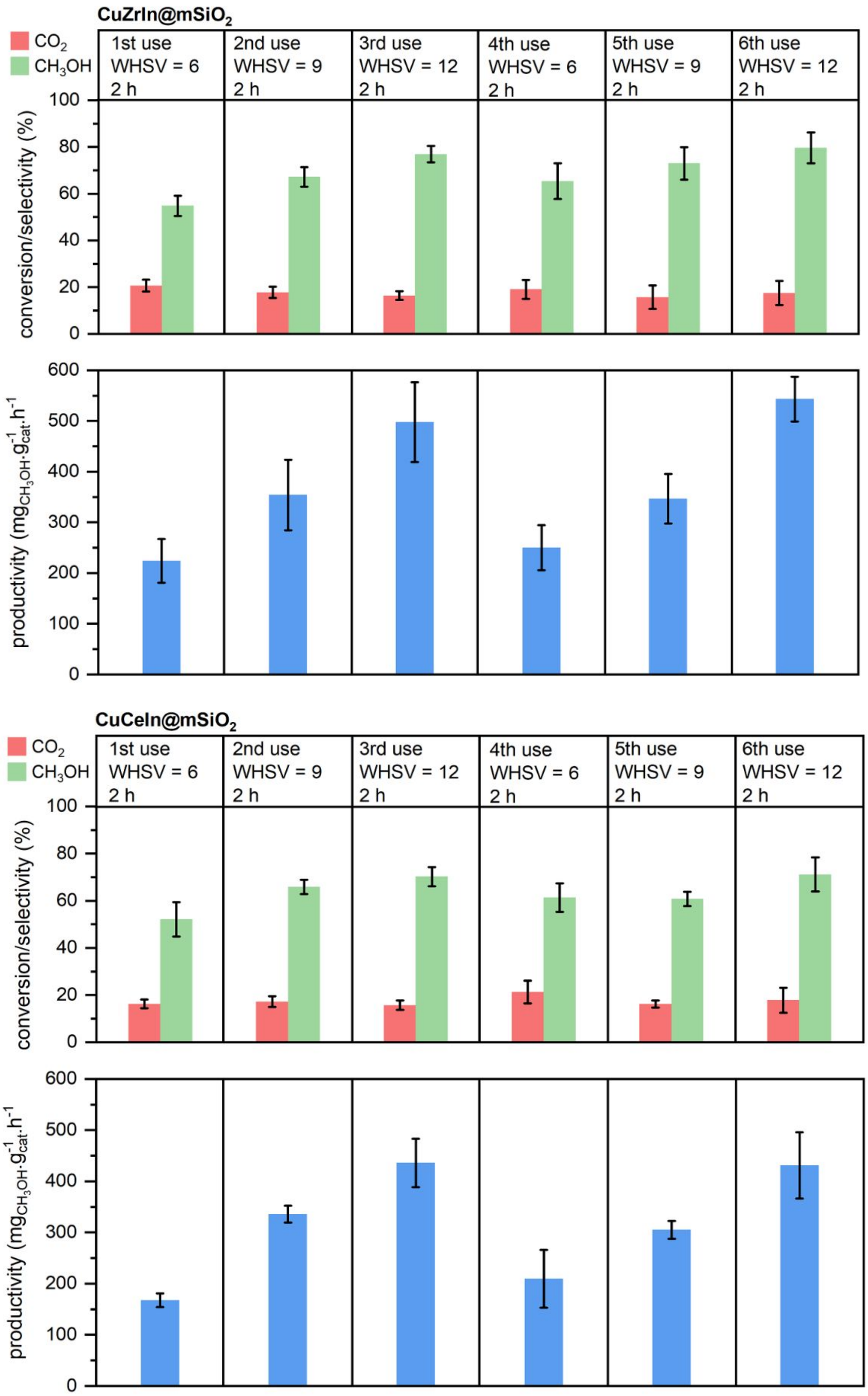

Figure S6. Catalytic activity in terms of CO_2 conversion, CH_3OH selectivity and productivity during reuse tests. Before each use catalysts were reduced in H_2 (30 mL.min^{-1}) for 1 h at 573 K. WHSV values in the figure are in L.g^{-1}.h^{-1}.

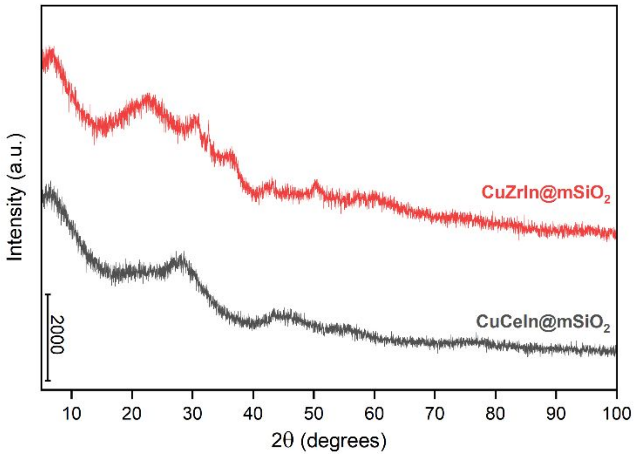

Figure S7. XRD diffraction patterns of spent catalysts.

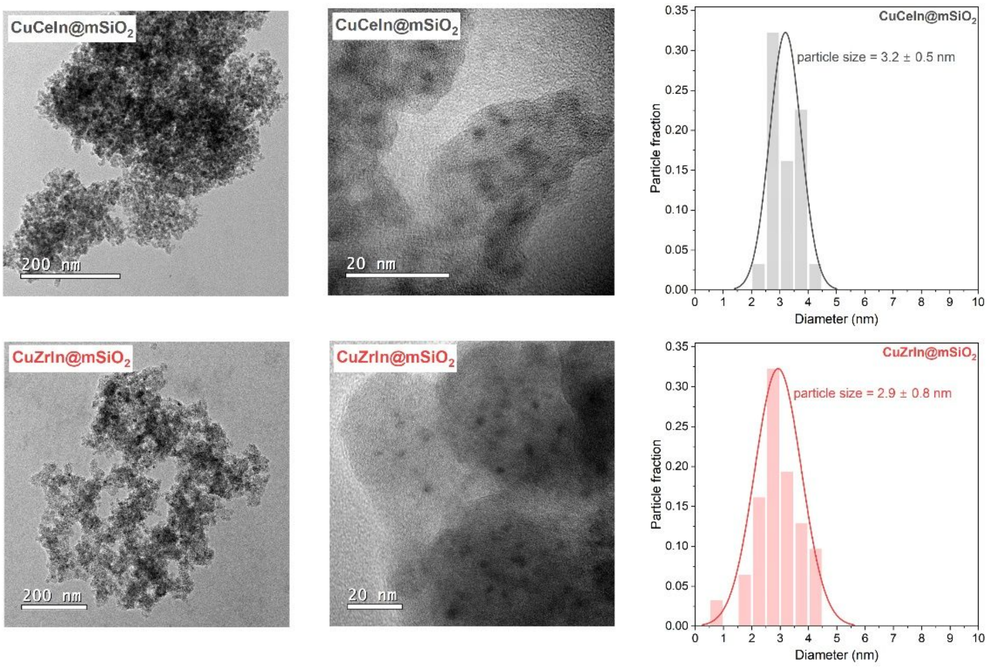

Figure S8. Transmission Electron Microscopy (TEM) images of spent catalysts.

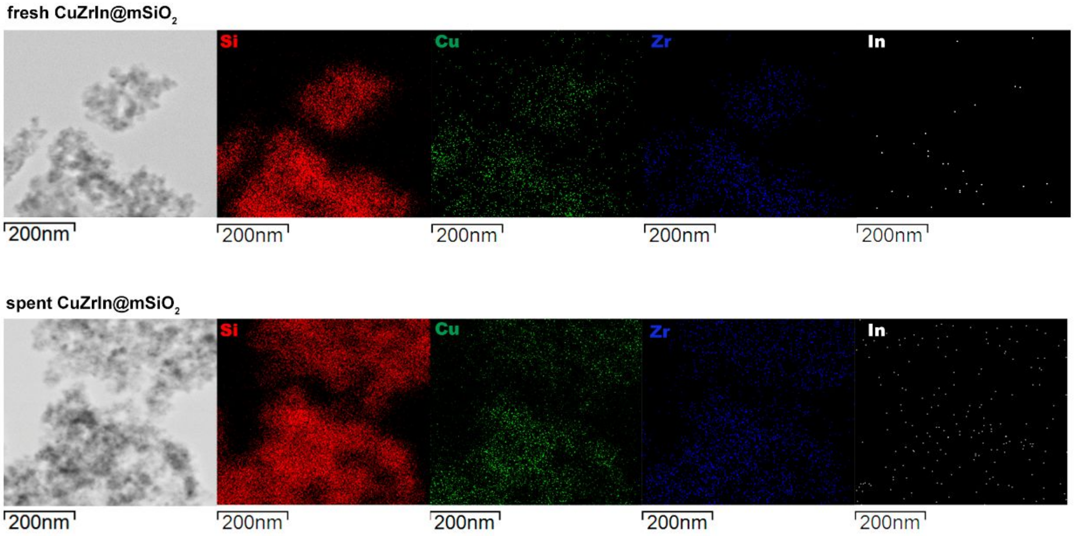

Figure S9. Elemental mappings obtained through Energy Dispersive Spectroscopy (EDS) of fresh and spent CuZrIn@$mSiO_2$ catalyst.

Supplementary Tables

Table S1. Metallic surface area and basicity normalized by the amount of active phases in catalysts.

**Catalyst**	**Specific basicity (mmol$_{CO2}$. g$_{CuZrCeIn.}$$^{-1}$)**	**Specific metallic surface area (m$_{Cu}$^{2}.g$_{CuZrCeIn.}$$^{-1}$)**
CuCeIn@mSiO$_2$	1.340	67.9
CuCeIn	0.242	10.5
CuZrIn@mSiO$_2$	0.866	94.2
CuZrIn	0.126	20.3

Table S2. Values defined for the minimum, maximum, average, minimum axial and maximum axial points of the pressure, temperature, and space velocity parameters.

Variable	Minimum point (-1)	Maximum point (+1)	Central point (0)	Minimum axis point (-1,68)	Maximum axis point (+1,68)
P (MPa)	2.0	3.0	2.5	1.7	3.3
T (K)	473	523	498	456	540
WHSV ($L.g^{-1}.h^{-1}$)	6.00	12.00	9.00	3.95	14.05

Table S3. Matrix of experiments defined by the Central Composite experimental planning methodology based on combinations between minimum, maximum, average, and axial points of each parameter.

Test	P (MPa)	T (K)	WHSV ($L.g^{-1}.h^{-1}$)
1	2.0	473	6.00
2	2.0	473	12.00
3	2.0	523	6.00
4	2.0	523	12.00
5	3.0	473	6.00
6	3.0	473	12.00
7	3.0	523	6.00
8	3.0	523	12.00
9	1.7	498	9.00
10	3.3	498	9.00
11	2.5	456	9.00
12	2.5	540	9.00
13	2.5	498	3.95
14	2.5	498	14.05
15	2.5	498	9.00
16	2.5	498	9.00
17	2.5	498	9.00

Table S4. Catalytic results for CuZrIn@mSiO$_2$ catalyst based on the experimental matrix of Table S4.

Test	P (MPa)	T (K)	WHSV ($L.g^{-1}.h^{-1}$)	X_{CO2} (%)	S_{CH3OH} (%)	S_{CO} (%)	S_{CH4} (%)	S_{C2H6} (%)
1	2.0	473	6.00	2.2	87.2	4.8	7.4	0.6
2	2.0	473	12.00	1.4	89.2	3.8	6.6	0.4
3	2.0	523	6.00	8.3	78.9	9.8	9.5	1.8
4	2.0	523	12.00	5.0	77.8	11.2	9.6	1.4
5	3.0	473	6.00	4.5	91.2	3.7	4.2	0.9
6	3.0	473	12.00	2.9	92.1	3.6	3.7	0.6
7	3.0	523	6.00	15.7	80.4	15.0	2.7	1.9
8	3.0	523	12.00	10.8	82.6	13.2	2.9	1.3
9	1.7	498	9.00	3.0	85.4	5.3	8.3	1.0
10	3.3	498	9.00	8.3	91.1	5.4	2.6	0.9
11	2.5	456	9.00	1.3	96.2	2.0	1.5	0.3
12	2.5	540	9.00	12.1	76.8	12.1	9.1	2.0
13	2.5	498	3.95	10.0	89.7	5.1	3.6	1.6
14	2.5	498	14.05	4.2	91.8	4.4	3.1	0.7
15	2.5	498	9.00	5.4	90.9	4.7	3.4	1.0
16	2.5	498	9.00	5.6	90.5	5.0	3.1	1.2
17	2.5	498	9.00	5.3	91.1	4.2	3.8	1.0

Table S5. Catalytic results for CuZrIn catalyst based on the experimental matrix of Table S4.

Test	P (MPa)	T (K)	WHSV ($L.g^{-1}.h^{-1}$)	X_{CO2} (%)	S_{CH3OH} (%)	S_{CO} (%)	S_{CH4} (%)	S_{C2H6} (%)
1	2.0	473	6.00	2.7	80.0	19.7	0.1	0.2
2	2.0	473	12.00	1.7	82.6	17.1	0.1	0.2
3	2.0	523	6.00	13.5	37.7	62.1	0.2	0.0
4	2.0	523	12.00	8.0	49.5	50.3	0.2	0.0
5	3.0	473	6.00	4.4	79.0	20.8	0.1	0.1
6	3.0	473	12.00	2.6	83.7	16.0	0.1	0.2
7	3.0	523	6.00	17.8	45.9	54.0	0.1	0.0
8	3.0	523	12.00	11.6	52.0	47.9	0.1	0.0
9	1.7	498	9.00	4.2	65.7	34.1	0.1	0.1
10	3.3	498	9.00	6.6	69.5	30.3	0.1	0.1
11	2.5	456	9.00	1.3	89.5	10.2	0.0	0.3
12	2.5	540	9.00	18.8	33.4	66.4	0.2	0.0
13	2.5	498	3.95	12.2	57.2	42.7	0.1	0.0
14	2.5	498	14.05	4.0	70.4	29.4	0.1	0.1
15	2.5	498	9.00	5.5	67.5	32.3	0.1	0.1
16	2.5	498	9.00	5.6	66.2	33.5	0.2	0.1
17	2.5	498	9.00	5.3	68.5	31.3	0.1	0.1

Table S6. Catalytic results for CuCeIn@mSiO$_2$ catalyst based on the experimental matrix of Table S4.

Test	P (MPa)	T (K)	WHSV ($L.g^{-1}.h^{-1}$)	X_{CO2} (%)	S_{CH3OH} (%)	S_{CO} (%)	S_{CH4} (%)	S_{C2H6} (%)
1	2.0	473	6.00	2.0	90.9	3.2	5.7	0.2
2	2.0	473	12.00	1.0	88.5	3.6	6.5	1.4
3	2.0	523	6.00	7.8	85.4	11.6	3.0	0.0
4	2.0	523	12.00	4.1	86.0	11.0	3.0	0.0
5	3.0	473	6.00	3.4	90.2	3.8	5.3	0.7
6	3.0	473	12.00	1.9	93.5	2.0	4.4	0.1
7	3.0	523	6.00	12.2	87.3	10.4	2.3	0.0
8	3.0	523	12.00	7.8	88.9	9.0	2.1	0.0
9	1.7	498	9.00	2.1	89.0	7.0	4.0	0.0
10	3.3	498	9.00	6.2	94.6	3.1	2.3	0.0
11	2.5	456	9.00	0.9	93.9	1.4	2.7	2.0
12	2.5	540	9.00	10.2	81.5	15.5	3.0	0.0
13	2.5	498	3.95	8.6	93.5	3.9	2.6	0.0
14	2.5	498	14.05	3.2	93.7	3.7	2.6	0.0
15	2.5	498	9.00	4.2	93.0	4.2	2.9	0.0
16	2.5	498	9.00	4.0	93.4	4.2	3.0	0.0
17	2.5	498	9.00	4.2	94.1	3.7	3.2	0.0

Table S7. Catalytic results for CuCeIn catalyst based on the experimental matrix of Table S4.

Test	P (MPa)	T (K)	WHSV ($L.g^{-1}.h^{-1}$)	X_{CO2} (%)	S_{CH3OH} (%)	S_{CO} (%)	S_{CH4} (%)	S_{C2H6} (%)
1	2.0	473	6.00	0.9	72.7	26.5	0.5	0.3
2	2.0	473	12.00	0.6	73.8	25.3	0.4	0.5
3	2.0	523	6.00	6.0	32.6	66.8	0.5	0.1
4	2.0	523	12.00	3.7	34.8	64.7	0.4	0.1
5	3.0	473	6.00	1.7	78.5	20.6	0.5	0.4
6	3.0	473	12.00	1.0	80.1	19.2	0.3	0.4
7	3.0	523	6.00	7.7	37.5	61.9	0.5	0.1
8	3.0	523	12.00	4.7	42.2	57.3	0.4	0.1
9	1.7	498	9.00	1.5	50.9	48.5	0.5	0.1
10	3.3	498	9.00	3.0	62.4	37.1	0.4	0.1
11	2.5	456	9.00	0.3	98.8	0.0	0.2	1.0
12	2.5	540	9.00	7.9	30.1	69.2	0.7	0.0
13	2.5	498	3.95	3.3	55.4	44.0	0.5	0.1
14	2.5	498	14.05	1.4	61.4	37.9	0.5	0.2
15	2.5	498	9.00	1.8	60.3	39.0	0.5	0.2
16	2.5	498	9.00	1.9	59.4	40.0	0.4	0.2
17	2.5	498	9.00	1.8	59.8	39.6	0.4	0.2

Table S8. Catalytic performance of Cu-based materials during CO_2 hydrogenation to methanol reported in the literature.

**Catalyst**	**Content (wt. %)**		**Reaction conditions**			**Catalytic performance**			**Ref.**
	**Cu**	**In**	**P (MPa)**	**T (K)**	**WHSV ($L.g_{cat}^{-1}.h^{-1}$)**	**X_{CO2} (%)**	**S_{MeOH} (%)**	**Space-time yield ($g_{MeOH}.g_{cat}^{-1}.h^{-1}$)**	
In_2O_3	-	41.0	4.0	603	15.0	7.1	39.7	0.118	1
Cu/In_2O_3	3.9	~39.5	5.0	573	6.0	~17.0	~45.0	0.140	2
Cu/CeO_2(sphere)	5.0	-	3.0	523	30.0	1.5	52.0	0.064	3
Cu/ZrO_2	5.0	-	0.1	493	75.0	0.53	19.8	0.022	4
Cu/ZrO_2	10	-	3.0	553	7.2	12.1	31.0	0.096	5
$Cu/In_2O_3/CeO_2$	4.6	0.9	3.0	483	7.2	~7.5	95	0.023	6
$Cu/In_2O_3/ZrO_2$	22.1	3.11	3.0	543	18.0	~16.0	~35.0	0.398	7
$Cu_{0.25}In_{0.75}Zr_{0.5}O$	17.9	45.5	2.5	523	18.0	1.5	79.7	0.075	8
$Cu@SiO_2$	8.0	-	3.0	553	7.5	6.5	54.2	0.077	9
$In_2O_3@SiO_2$	-	6.6	3.0	553	7.5	4.3	89.0	0.082	9
$Cu/In_2O_3@SiO_2$	8.0	6.6	3.0	553	7.5	12.5	78.2	0.210	9
$Cu/ZnO@mSiO_2$	11.7	-	5.0	523	6.0	9.8	66.6	0.136	10
$Cu\text{-}CeO_2\text{-}ZnO/MV\mathit{m}SiO_2$	4.0	-	3.0	553	18.0	-	~18.0	0.046	11
$Cu\text{-}ZrO_2\text{-}ZnO/MV\mathit{m}SiO_2$	4.5	-	3.0	553	18.0	-	~27.0	0.059	11
$Cu/ZrO_2/In_2O_3@mSiO_2$	6.5	1.1	3.0	523	12.0	10.8	82.6	0.345	This work
$Cu/CeO_2/In_2O_3@mSiO_2$	5.6	0.8	3.0	523	12.0	7.8	88.9	0.268	This work

Table S9. Main effects in CH_3OH selectivity by changing pressure, temperature and space velocity and the combination of these variables in CuZrIn@mSiO$_{2+}$ catalyst.

Variable	Effect	Error	Standardized effect (t)	Significant
Pressure (X_1)	3.3370	0.1653	20.1827	yes
Temperature (X_2)	-10.6359	0.1653	-64.3284	yes
GHSV (X_3)	1.1030	0.1653	6.6712	yes
$X_1 \times X_2$	-0.1500	0.2160	-0.6944	no
$X_1 \times X_3$	0.5500	0.2160	2.5460	no
$X_2 \times X_3$	-0.4500	0.2160	-2.0831	no

Table S10. Main effects in CH_3OH selectivity by changing pressure, temperature and space velocity and the combination of these variables in CuZrIn catalyst.

Variable	Effect	Error	Standardized effect (t)	Significant
Pressure (X_1)	2.5175	0.6241	4.0336	no
Temperature (X_2)	-34.3488	0.6241	-55.0341	yes
GHSV (X_3)	6.9415	0.6241	11.1218	yes
$X_1 \times X_2$	2.6500	0.8155	3.2496	no
$X_1 \times X_3$	-0.9000	0.8155	-1.1037	no
$X_2 \times X_3$	2.6500	0.8155	3.2496	no

Table S11. Main effects in CH_3OH selectivity by changing pressure, temperature and space velocity and the combination of these variables in CuCeIn@mSiO$_2$ catalyst.

Variable	Effect	Error	Standardized effect (t)	Significant
Pressure (X_1)	2.7119	0.3013	8.9999	yes
Temperature (X_2)	-5.3240	0.3013	-17.6685	yes
GHSV (X_3)	0.5032	0.3013	1.6701	no
$X_1 \times X_2$	0.1250	0.3937	0.3175	no
$X_1 \times X_3$	1.6750	0.3937	4.2545	no
$X_2 \times X_3$	0.3250	0.3937	0.8255	no

Table S12. Main effects in CH_3OH selectivity by changing pressure, temperature and space velocity and the combination of these variables in CuCeIn.

Variable	Effect	Error	Standardized effect (t)	Significant
Pressure (X_1)	6.4057	0.2440	26.2485	yes
Temperature (X_2)	-40.0589	0.2440	-164.1496	yes
GHSV (X_3)	2.8836	0.2440	11.8163	yes
$X_1 \times X_2$	0.0500	0.3189	0.1568	no
$X_1 \times X_3$	0.7500	0.3189	2.3522	no
$X_2 \times X_3$	1.0500	0.3189	3.2931	no

Table S13. Main effects in CO_2 conversion by changing pressure, temperature and space velocity and the combination of these variables in CuZrIn@mSiO$_{2+}$ catalyst.

Variable	Effect	Error	Standardized effect (t)	Significant
Pressure (X_1)	3.7949	0.0827	45.9053	yes
Temperature (X_2)	6.8776	0.0827	83.1947	yes
GHSV (X_3)	-2.9808	0.0827	-36.0574	yes
$X_1 \times X_2$	2.3500	0.1080	21.7568	yes
$X_1 \times X_3$	-0.6000	0.1080	-5.5549	yes
$X_2 \times X_3$	-1.4500	0.1080	-13.4244	yes

Table S14. Main effects in CO_2 conversion by changing pressure, temperature and space velocity and the combination of these variables in CuZrIn catalyst.

Variable	Effect	Error	Standardized effect (t)	Significant
Pressure (X_1)	2.1288	0.0827	25.7508	yes
Temperature (X_2)	10.0948	0.0827	122.1106	yes
GHSV (X_3)	-4.1431	0.0827	-50.1164	yes
$X_1 \times X_2$	1.3250	0.1080	12.2671	yes
$X_1 \times X_3$	-0.3750	0.1080	-3.4718	no
$X_2 \times X_3$	-2.2250	0.1080	-20.5995	yes

Table S15. Main effects in CO_2 conversion by changing pressure, temperature and space velocity and the combination of these variables in CuCeIn@mSiO_{2+} catalyst.

Variable	Effect	Error	Standardized effect (t)	Significant
Pressure (X_1)	2.5328	0.0625	40.5308	yes
Temperature (X_2)	5.7467	0.0625	91.9585	yes
GHSV (X_3)	-2.8823	0.0625	-46.1230	yes
$X_1 \times X_2$	1.4500	0.0816	17.7588	yes
$X_1 \times X_3$	-0.3000	0.0816	-3.6742	no
$X_2 \times X_3$	-1.4000	0.0816	-17.1464	yes

Table S16. Main effects in CO_2 conversion by changing pressure, temperature and space velocity and the combination of these variables in CuCeIn catalyst.

Variable	Effect	Error	Standardized effect (t)	Significant
Pressure (X_1)	0.9406	0.0312	30.1025	yes
Temperature (X_2)	4.4932	0.0312	143.8016	yes
GHSV (X_3)	-1.3906	0.0312	-44.5040	yes
$X_1 \times X_2$	0.3750	0.0408	9.1856	yes
$X_1 \times X_3$	-0.2750	0.0408	-6.7361	yes
$X_2 \times X_3$	-1.0750	0.0408	-26.3320	yes

Table S17. Main effects in CH_3OH productivity by changing pressure, temperature and space velocity and the combination of these variables in CuZrIn@mSiO_{2+} catalyst.

Variable	Effect	Error	Standardized effect (t)	Significant
Pressure (X_1)	95.6923	2.0496	46.6872	yes

Temperature (X_2)	145.0938	2.0496	70.7895	yes
GHSV (X_3)	38.2783	2.0496	18.6755	yes
$X_1 \times X_2$	53.7000	2.6780	20.0523	yes
$X_1 \times X_3$	22.4500	2.6780	8.3831	yes
$X_2 \times X_3$	22.4000	2.6780	8.3645	yes

Table S18. Main effects in CH_3OH productivity by changing pressure, temperature and space velocity and the combination of these variables in CuZrIn catalyst.

Variable	Effect	Error	Standardized effect (t)	Significant
Pressure (X_1)	41.6253	0.7348	56.6501	yes
Temperature (X_2)	94.4695	0.7348	128.5686	yes
GHSV (X_3)	32.9191	0.7348	44.8013	yes
$X_1 \times X_2$	21.1250	0.9600	22.0044	yes
$X_1 \times X_3$	6.2750	0.9600	6.5362	yes
$X_2 \times X_3$	25.1750	0.9600	26.2230	yes

Table S19. Main effects in CH_3OH productivity by changing pressure, temperature and space velocity and the combination of these variables in CuCeIn@mSiO$_{2+}$ catalyst.

Variable	Effect	Error	Standardized effect (t)	Significant
Pressure (X_1)	67.7737	1.7673	38.3495	yes
Temperature (X_2)	132.7604	1.7673	75.1220	yes
GHSV (X_3)	19.6930	1.7673	11.1432	yes
$X_1 \times X_2$	37.6250	2.3090	16.2946	yes
$X_1 \times X_3$	16.2750	2.3090	7.0484	yes
$X_2 \times X_3$	15.3750	2.3090	6.6586	yes

Table S20. Main effects in CH_3OH productivity by changing pressure, temperature and space velocity and the combination of these variables in CuCeIn catalyst.

Variable	Effect	Error	Standardized effect (t)	Significant
Pressure (X_1)	18.4502	0.4296	42.9512	yes
Temperature (X_2)	34.4414	0.4296	80.1780	yes
GHSV (X_3)	10.0515	0.4296	23.3995	yes
$X_1 \times X_2$	4.5000	0.5612	8.0178	yes
$X_1 \times X_3$	2.4000	0.5612	4.2762	no
$X_2 \times X_3$	5.8000	0.5612	10.3341	yes

Table S21. Analysis of variance (ANOVA) for the quadratic model applied using the catalyst CuZrIn@mSiO$_2$ and the CH$_3$OH selectivity results from central composite experimental design.

Source of variation	Sum of squares	Degrees of freedom	Mean sum of squares	F-ratio
Regression	501.2619	9	55.6958	9.2401
Residual	42.1934	7	6.0276	
Lack-of-fit	42.0067	5	8.4013	90.0145
Pure error	0.1867	2	0.0933	
Total	543.4553	16		
R^2	0.9224			
Adjusted R^2	0.9997			

Table S22. Analysis of variance (ANOVA) for the quadratic model applied using the catalyst CuZrIn and the CH$_3$OH selectivity results from central composite experimental design.

Source of variation	Sum of squares	Degrees of freedom	Mean sum of squares	F-ratio
Regression	$4,2819 \times 10^3$	6	713,6461	159,3933
Residual	44,7727	10	4,4773	-
Lack-of-fit	42,1127	8	5,2641	3,9580
Pure error	2,6600	2	1,3300	-
Total	$4,3266 \times 10^3$	16		
R^2	0,9897			
Adjusted R^2	0,9994			

Table S23. Analysis of variance (ANOVA) for the quadratic model applied using the catalyst CuCeIn@mSiO$_2$ and the CH$_3$OH selectivity results from central composite experimental design.

Source of variation	Sum of squares	Degrees of freedom	Mean sum of squares	F-ratio
Regression	202.5763	9	22.5085	5.5986
Residual	28.1425	7	4.0204	
Lack-of-fit	27.5225	5	5.5045	17.7565
Pure error	0.6200	2	0.3100	
Total	230.7188	16		
R^2	0.8780			
Adjusted R^2	0.9973			

Table S24. Analysis of variance (ANOVA) for the quadratic model applied using the catalyst CuCeIn and the CH_3OH selectivity results from central composite experimental design.

**Source of variation**	**Sum of squares**	**Degrees of freedom**	**Mean sum of squares**	**F-ratio**
Regression	$5.7291x10^3$	9	636.5698	114.6009
Residual	38.8827	7	5.5547	
Lack-of-fit	38.4760	5	7.6952	37.8453
Pure error	0.4067	2	0.2033	
Total	$5.7680x10^3$	16		
R^2	0.9933			
Adjusted R^2	0.9846			

Table S25. Analysis of variance (ANOVA) for the quadratic model applied using the catalyst CuZrIn@mSiO$_2$ and the CO_2 conversion results from central composite experimental design.

**Source of variation**	**Sum of squares**	**Degrees of freedom**	**Mean sum of squares**	**F-ratio**
Regression	261.4983	9	29.0554	89.1847
Residual	2.2805	7	0.3258	
Lack-of-fit	2.2339	5	0.4468	19.1473
Pure error	0.0467	2	0.0233	
Total	263.7788	16		
R^2	0.9914			
Adjusted R^2	0.9802			

Table S26. Analysis of variance (ANOVA) for the quadratic model applied using the catalyst CuZrIn and the CO_2 conversion results from central composite experimental design.

**Source of variation**	**Sum of squares**	**Degrees of freedom**	**Mean sum of squares**	**F-ratio**
Regression	470.9974	9	52.3330	126.2067
Residual	2.9026	7	0.4147	
Lack-of-fit	2.8560	5	0.5712	24.4797
Pure error	0.0467	2	0.0233	
Total	473.9000	16		
R^2	0.9939			
Adjusted R^2	0.9860			

Table S27. Analysis of variance (ANOVA) for the quadratic model applied using the catalyst CuCeIn@mSiO_2 and the CO_2 conversion results from central composite experimental design.

Source of variation	Sum of squares	Degrees of freedom	Mean sum of squares	F-ratio
Regression	176.9846	9	19.6650	225.4020
Residual	0.6107	7	0.0872	
Lack-of-fit	0.5840	5	0.1168	8.7606
Pure error	0.0267	2	0.0133	
Total	177.5953	16		
R^2	0.9966			
Adjusted R^2	0.9921			

Table S28. Analysis of variance (ANOVA) for the quadratic model applied using the catalyst CuCeIn and the CO_2 conversion results from central composite experimental design.

Source of variation	Sum of squares	Degrees of freedom	Mean sum of squares	F-ratio
Regression	89.9078	9	9.9898	134.0642
Residual	0.5216	7	0.0745	
Lack-of-fit	0.5149	5	0.1030	30.8962
Pure error	0.0067	2	0.0033	
Total	90.4294	16		
R^2	0.9942			
Adjusted R^2	0.9868			

Table S29. Analysis of variance (ANOVA) for the quadratic model applied using the catalyst CuZrIn@mSiO_2 and the CH_3OH productivity results from central composite experimental design.

Source of variation	Sum of squares	Degrees of freedom	Mean sum of squares	F-ratio
Regression	1.1600×10^5	9	1.2889×10^4	89.4596
Residual	1.0085×10^3	7	144.0760	
Lack-of-fit	979.8452	5	195.9690	13.6627
Pure error	28.6867	2	14.3433	
Total	1.1701×10^5	16		
R^2	0.9914			
Adjusted R^2	0.9803			

Table S30. Analysis of variance (ANOVA) for the quadratic model applied using the catalyst CuZrIn and the CH_3OH productivity results from central composite experimental design.

Source of variation	Sum of squares	Degrees of freedom	Mean sum of squares	F-ratio
Regression	$4.2352x10^4$	9	4.7058e+03	50.2073
Residual	656.0920	7	93.7274	
Lack-of-fit	652.4054	5	130.4811	70.7854
Pure error	3.6867	2	1.8433	
Total	$4.3008x10^4$	16		
R^2	0.9847			
Adjusted R^2	0.9651			

Table S31. Analysis of variance (ANOVA) for the quadratic model applied using the catalyst CuCeIn@$mSiO_2$ and the CH_3OH productivity results from central composite experimental design.

Source of variation	Sum of squares	Degrees of freedom	Mean sum of squares	F-ratio
Regression	$8.1584x10^4$	9	9.0649e+03	165.7810
Residual	382.7590	7	54.6799	
Lack-of-fit	361.4324	5	72.2865	6.7790
Pure error	21.3267	2	10.6633	
Total	$8.1967x10^4$	16		
R^2	0.9953			
Adjusted R^2	0.9893			

Table S32. Analysis of variance (ANOVA) for the quadratic model applied using the catalyst CuCeIn and the CH_3OH productivity results from central composite experimental design.

Source of variation	Sum of squares	Degrees of freedom	Mean sum of squares	F-ratio
Regression	$5.8316x10^3$	9	647.9538	150.0850
Residual	30.2207	7	4.3172	
Lack-of-fit	28.9607	5	5.7921	9.1939
Pure error	1.2600	2	0.6300	
Total	$5.8618x10^3$	16		
R^2	0.9948			
Adjusted R^2	0.9882			

Supplementary equations

$S_{CH_3OH}(CuCeIn@SiO_2) = (12.4488 \times P) + (3.5462 \times T) - (1.9386 \times WHSV) + (0.0050 \times P \times T) + (0.5583 \times P \times WHSV) + (0.0022 \times T \times WHSV) - (3.4504 \times P^2) - (0.0037 \times T^2) - (0.0251 \times WHSV^2) - 774.0233$ (S6)

$X_{CO_2}(CuCeIn@SiO_2) = -(24.5047 \times P) - (0.6594 \times T) + (3.2748 \times WHSV) + (0.0580 \times P \times T) - (0.1000 \times P \times WHSV) - (0.0093 \times T \times WHSV) - (0.1893 \times P^2) + (7.1624 \times 10^{-4} \times T^2) + (0.0635 \times WHSV^2) + 154.6219$ (S7)

$STY_{CH_3OH}(CuCeIn@SiO_2) = -(714.0929 \times P) - (12.3539 \times T) - (64.5595 \times WHSV) + (1.5050 \times P \times T) + (5.4250 \times P \times WHSV) + (0.1025 \times T \times WHSV) - (3.2897 \times P^2) + (0.0104 \times T^2) + (0.1797 \times WHSV^2) + 3610.4$ (S8)

$S_{CH_3OH}(CuCeIn) = (40.3586 \times P) - (2.2999 \times T) - (1.1479 \times WHSV) + (0.0020 \times P \times T) + (0.2500 \times P \times WHSV) + (0.0070 \times T \times WHSV) - (7.4398 \times P^2) + (0.0014 \times T^2) - (0.1379 \times WHSV^2) + 776.7542$ (S9)

$X_{CO_2}(CuCeIn) = -(10.0839 \times P) - (1.2744 \times T) + (3.0577 \times WHSV) + (0.0150 \times P \times T) - (0.0917 \times P \times WHSV) - (0.0072 \times T \times WHSV) + (0.8759 \times P^2) + (0.0014 \times T^2) + (0.0283 \times WHSV^2) + 295.4812$ (S10)

$STY_{CH_3OH}(CuCeIn) = -(133.0658 \times P) - (4.7756 \times T) - (19.9925 \times WHSV) + (0.1800 \times P \times T) + (0.8000 \times P \times WHSV) + (0.0387 \times T \times WHSV) + (10.9352 \times P^2) + (0.0047 \times T^2) + (0.0229 \times WHSV^2) + 1275.0$ (S11)

$S_{CH_3OH}(CuZrIn@SiO_2) = (38.8670 \times P) + (3.5397 \times T) + (2.8709 \times WHSV) - (0.0060 \times P \times T) + (0.1833 \times P \times WHSV) - (0.0030 \times T \times WHSV) - (6.8384 \times P^2) - (0.0037 \times T^2) - (0.0917 \times WHSV^2) - 803.7628$ (S12)

$X_{CO_2}(CuZrIn@SiO_2) = -(41.8743 \times P) - (0.6544 \times T) + (3.7262 \times WHSV) + (0.0940 \times P \times T) - (0.2000 \times P \times WHSV) - (0.0097 \times T \times WHSV) + (0.1314 \times P^2) + (6.4655 \times 10^{-4} \times T^2) + (0.0606 \times WHSV^2) + 167.2108$ (S13)

$STY_{CH_3OH}(CuZrIn@SiO_2) = -(1037.0 \times P) - (6.9234 \times T) - (84.4930 \times WHSV) + (2.1417 \times P \times T) + (7.4917 \times P \times WHSV) + (0.1492 \times T \times WHSV) - (0.8133 \times P^2) + (0.0031 \times T^2) - (0.1188 \times WHSV^2) + 2673.8$ (S14)

$$S_{CH_3OH}(CuZrIn) = -(47.6781 \times P) + (2.3455 \times T) - (4.2148 \times WHSV) + (0.1060 \times P \times T) - (0.3000 \times P \times WHSV) + (0.0177 \times T \times WHSV) + (0.0215 \times P^2) - (0.0035 \times T^2) - (0.1487 \times WHSV^2) - 275.3418 \quad \text{(S15)}$$

$$X_{CO_2}(CuZrIn) = -(21.5096 \times P) - (2.3062 \times T) + (5.2389 \times WHSV) + (0.0530 \times P \times T) - (0.1250 \times P \times WHSV) - (0.0148 \times T \times WHSV) - (0.2782 \times P^2) + (0.0025 \times T^2) + (0.0983 \times WHSV^2) + 533.5096 \quad \text{(S16)}$$

$$STY_{CH_3OH}(CuZrIn@SiO_2) = -(412.3320 \times P) - (3.6636 \times T) - (85.9226 \times WHSV) + (0.8450 \times P \times T) + (2.0917 \times P \times WHSV) + (0.1678 \times T \times WHSV) + (2.8645 \times P^2) + (0.0019 \times T^2) + (0.1444 \times WHSV^2) + 1373.8 \quad \text{(S17)}$$

References

(1) Sun, K.; Fan, Z.; Ye, J.; Yan, J.; Ge, Q.; Li, Y.; He, W.; Yang, W.; Liu, C. Hydrogenation of CO2 to Methanol over In2O3 Catalyst. *Journal of CO2 Utilization* **2015**, *12*, 1–6. https://doi.org/https://doi.org/10.1016/j.jcou.2015.09.002.

(2) Zou, R.; Shen, C.; Sun, K.; Ma, X.; Li, Z.; Li, M.; Liu, C.-J. CO2 Hydrogenation to Methanol over the Copper Promoted In2O3 Catalyst. *Journal of Energy Chemistry* **2024**, *93*, 135–145. https://doi.org/https://doi.org/10.1016/j.jechem.2024.01.027.

(3) Zhu, J.; Su, Y.; Chai, J.; Muravev, V.; Kosinov, N.; Hensen, E. J. M. Mechanism and Nature of Active Sites for Methanol Synthesis from CO/CO2 on Cu/CeO2. *ACS Catal* **2020**, *10* (19), 11532–11544. https://doi.org/10.1021/acscatal.0c02909.

(4) Kattel, S.; Yan, B.; Yang, Y.; Chen, J. G.; Liu, P. Optimizing Binding Energies of Key Intermediates for CO2 Hydrogenation to Methanol over Oxide-Supported Copper. *J Am Chem Soc* **2016**, *138* (38), 12440–12450. https://doi.org/10.1021/jacs.6b05791.

(5) Witoon, T.; Chalorngtham, J.; Dumrongbunditkul, P.; Chareonpanich, M.; Limtrakul, J. CO2 Hydrogenation to Methanol over Cu/ZrO2 Catalysts: Effects of Zirconia Phases. *Chemical Engineering Journal* **2016**, *293*, 327–336. https://doi.org/https://doi.org/10.1016/j.cej.2016.02.069.

(6) Sharma, S. K.; Paul, B.; Pal, R. S.; Bhanja, P.; Banerjee, A.; Samanta, C.; Bal, R. Influence of Indium as a Promoter on the Stability and Selectivity of the Nanocrystalline Cu/CeO2 Catalyst for CO2 Hydrogenation to Methanol. *ACS Appl Mater Interfaces* **2021**, *13* (24), 28201–28213. https://doi.org/10.1021/acsami.1c05586.

(7) Zhang, G.; Fan, G.; Yang, L.; Li, F. Tuning Surface-Interface Structures of ZrO2 Supported Copper Catalysts by in Situ Introduction of Indium to Promote CO2 Hydrogenation to Methanol. *Appl Catal A Gen* **2020**, *605*, 117805. https://doi.org/https://doi.org/10.1016/j.apcata.2020.117805.

(8) Yao, L.; Shen, X.; Pan, Y.; Peng, Z. Synergy between Active Sites of Cu-In-Zr-O Catalyst in CO2 Hydrogenation to Methanol. *J Catal* **2019**, *372*, 74–85. https://doi.org/https://doi.org/10.1016/j.jcat.2019.02.021.

(9) Shi, Z.; Tan, Q.; Wu, D. A Novel Core–Shell Structured CuIn@SiO2 Catalyst for CO2 Hydrogenation to Methanol. *AIChE Journal* **2019**, *65* (3), 1047–1058. https://doi.org/https://doi.org/10.1002/aic.16490.

(10) Yang, H.; Gao, P.; Zhang, C.; Zhong, L.; Li, X.; Wang, S.; Wang, H.; Wei, W.; Sun, Y. Core–Shell Structured Cu@m-SiO2 and Cu/ZnO@m-SiO2 Catalysts for Methanol Synthesis from CO2 Hydrogenation. *Catal Commun* **2016**, *84*, 56–60. https://doi.org/https://doi.org/10.1016/j.catcom.2016.06.010.

(11) Kosari, M.; Anjum, U.; Xi, S.; Lim, A. M. H.; Seayad, A. M.; Raj, E. A. J.; Kozlov, S. M.; Borgna, A.; Zeng, H. C. Revamping SiO2 Spheres by Core–Shell Porosity Endowment to Construct a Mazelike Nanoreactor for Enhanced Catalysis in CO2 Hydrogenation to Methanol. *Adv Funct Mater* **2021**, *31* (47), 2102896. https://doi.org/https://doi.org/10.1002/adfm.202102896.
